# AUF1-mediated inhibition of autophagic lysosomal degradation contributes to CagA stability and *Helicobacter pylori*-induced inflammation

**DOI:** 10.1080/19490976.2024.2382766

**Published:** 2024-07-28

**Authors:** Huiling Zheng, Ting Zhang, Jing Zhang, Jing Ning, Weiwei Fu, Ye Wang, Yanyan Shi, Guochao Wei, Jing Zhang, Xiangmei Chen, Shigang Ding

**Affiliations:** a Department of Gastroenterology, Peking University Third Hospital, Beijing, China; b Beijing Key Laboratory for Helicobacter Pylori Infection and Upper Gastrointestinal Diseases (BZ0371), Beijing, China; c Department of Microbiology and Infectious Disease Center, School of Basic Medical Sciences, Peking University Health Science Center, Beijing, China; d Department of Laboratory Medicine, Beijing Tongren Hospital, Capital Medical University, Beijing, China; e Research Center of Clinical Epidemiology, Peking University Third Hospital, Beijing, P.R. China

**Keywords:** CagA degradation, AUF1, *Helicobacter pylori*-gastritis

## Abstract

CagA, a virulence factor of *Helicobacter pylori* (*H. pylori*), is known to drive inflammation in gastric epithelial cells and is typically degraded through autophagy. However, the molecular mechanism by which CagA evades autophagy-mediated degradation remains elusive. This study found that *H. pylori* inhibits autophagic flux by upregulating the expression of AU-rich element RNA-binding factor 1 (AUF1). We confirmed that AUF1 does not affect autophagy initiation but instead hampers lysosomal clearance, as evidenced by treatments with 3-MA, CQ and BafA1. Upregulated AUF1 stabilizes CagA protein levels by inhibiting the autolysosomal degradation of intracellular CagA in *H. pylori*-infected gastric epithelial cells. Knocking down AUF1 promotes CagA degradation, an effect that can be reversed by the lysosome inhibitor BafA1 and CQ. Transcriptome analysis of AUF1-knockdown gastric epithelial cells infected with *H. pylori* indicated that AUF1 regulates the expression of lysosomal-associated hydrolase genes, specifically CTSD, to inhibit autolysosomal degradation. Moreover, we observed that knockdown of AUF1 enhanced the stability of CTSD mRNA and identified AUF1 binding to the 3’UTR region of CTSD mRNA. AUF1-mediated downregulation of CTSD expression contributes to CagA stability, and AUF1 overexpression leads to an increase in CagA levels in exosomes, thus promoting extracellular inflammation. In clinical gastric mucosa, the expression of AUF1 and its cytoplasmic translocation are associated with *H. pylori*-associated gastritis, with CagA being necessary for the translocation of AUF1 into the cytoplasm. Our findings suggest that AUF1 is a novel host-positive regulator of CagA, and dysregulation of AUF1 expression increases the risk of *H. pylori*-associated gastritis.

## Introduction

*Helicobacter pylori* (*H. pylori*), classified as a class I carcinogen by the World Health Organization, is the leading risk factor for non-cardia gastric cancer, with approximately nine in 10 cases attributed to *H. pylori* infection^1–3^. Although the global prevalence of *H. pylori* infection has declined during the last 3 decades in adults, about 40% of the population is still affected by *H. pylori* infection.^4^ Coupled with rising antibiotic resistance, *H. pylori* infection remains a significant public health concern.^4–6^ The pathogenesis of *H. pylori*–related diseases is known to involve a complex interplay between host factors and bacterial virulence factors, leading to various conditions such as chronic superficial gastritis, atrophic gastritis, intestinal metaplasia, dysplasia and gastric cancer.^7,8^ Therefore, understanding the interaction between host factors and *H. pylori* virulence factors may provide new insights into the development of innovative therapies for *H. pylori*–related diseases.

Cytotoxin-associated gene A (CagA) is a well-studied *H. pylori* virulence factor that plays a crucial role in the pathogenesis of gastric diseases. As an effector of the type IV secretion system (T4SS), CagA is directly injected into gastric epithelial cells, where it will undergo tyrosine phosphorylation at EPIYA-motifs and then regulates multiple host signaling proteins in a phosphorylation-dependent or -independent fashion.^9,10^ Translocated CagA not only aggravates gastric mucosal inflammation via activating NF-κB pathways but also disrupts various cellular processes such as cell proliferation, migration, transformation, cell polarity, and apoptosis.^11,12^ It has been reported that some of CagA protein entering host cells are degraded through the autophagy-lysosomal pathway,^13,14^ while others are secreted through the exosome pathway, leading to extragastric disorders.^15,16^

Autophagy plays a crucial role in the degradation of intracellular proteins or organelles of host cells, and serves as a pivotal defense mechanism of innate immune system against pathogen invasion.^17^ The autophagic degradation of CagA in *H. pylori*-infected host cells serves as a protective strategy employed by the host to protect itself against CagA-induced damage. However, *H. pylori* itself employed host factors to inhibit autophagy and evade CagA degradation, thereby establishing persistent infection in the hostile gastric environment. One study has shown that CAPZA1, a negative regulator of autolysosome formation, is upregulated in *H. pylori*-infected gastric mucosa. This upregulation prevents the autophagic degradation of CagA, thereby increasing the risk of gastric carcinogenesis.^14^ Nonetheless, the precise mechanism underlying the interaction between host factors and CagA remains elusive.

The AU-rich element (ARE) RNA-binding factor 1 (AUF1), also known as heterogeneous nuclear ribonucleoprotein D (hnRNPD), is a well-studied protein that binds to AU-rich sequences in the 3’ untranslated regions (UTRs) of mRNAs. AUF1 has four different isoforms (p37, p40, p42, and p45) and its RNA recognition domains enable it to regulate mRNA degradation.^18–20^ AUF1 has been shown to be involved in autophagy by regulating the stability of BCL-2 mRNA in macrophages and DDIT4 mRNA in breast cancer cells.^21,22^ Our previous study demonstrated that *H. pylori* infection can upregulate AUF1 expression.^23^ However, the role of AUF1 on autophagy in gastric epithelial cells remains uncertain and it is also unclear whether upregulated AUF1 acts as a host factor that regulates the autophagic degradation of CagA in *H. pylori* infection.

In this study, we aimed to investigate the role of AUF1 on the autophagic degradation of CagA in gastric epithelial cells, as well as its underlying mechanism and clinical implications. Our findings revealed that AUF1 plays a significant role in promoting *H. pylori*-associated gastritis by inhibiting the autolysosomal degradation of CagA. These results suggest that AUF1 functions as a novel host-positive regulator of CagA, shedding light on its potential clinical significance.

## Results

### H. pylori inhibits autophagic flux through AUF1

**Figure 1.**
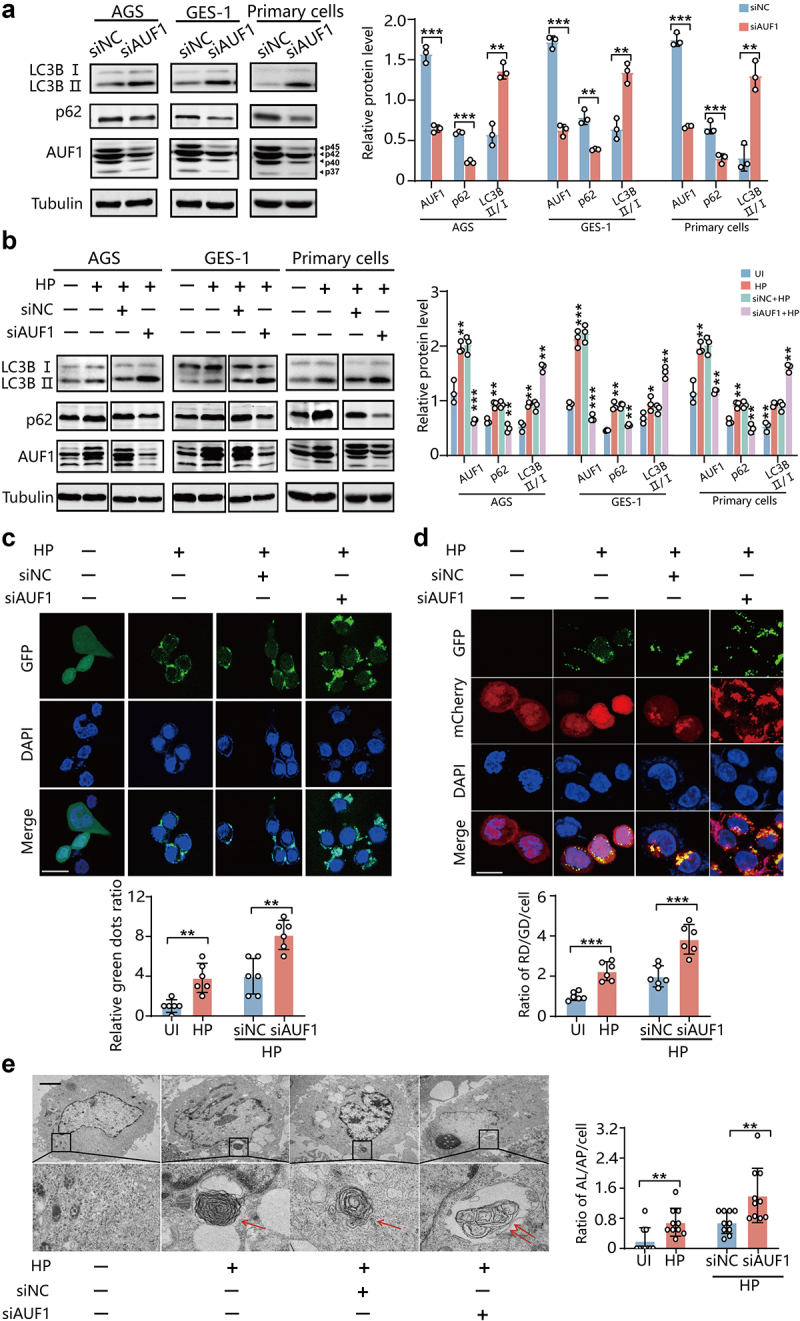
*H. pylori* inhibits autophagic flux through AUF1. (a) LC3B and p62 expression was analyzed by Western blot assays in AGS, GES-1 cells and human primary gastric epithelial cells with AUF1 knockdown. Gray values of LC3B, p62 and AUF1 band were analyzed by ImageJ. LC3B, p62 and AUF1/tubulin gray ratio was quantified, all the quantitative data are presented as means ± SD from three independent experiments. **p < .01, ***p < .001. (b) After AUF1 knockdown for 24 h, AGS, GES-1 and human primary gastric epithelial cells were subsequently infected with *H. pylori*. LC3B and p62 expression was analyzed by Western blot assays in *H.* *pylori* -infected or un-infected cells, as well as *H. pylori* -infected cells with control or AUF1 siRNA. Gray values of LC3B, p62 and AUF1 band were analyzed by ImageJ. LC3B, p62 and AUF1/tubulin gray ratio was quantified, all the quantitative data are presented as means ± SD from three independent experiments. *p < .05, **p < .01, ***p < .001. (c) The representative images illustrate GFP- LC3 reporter signals by if assays in *H. pylori* -infected (HP) or un-infected (UI) AGS cells, as well as *H. pylori*-infected AGS with control or AUF1 siRNA. The experiment was independently repeated three times. Scale bar 25 μm. Immunofluorescence intensity of green dots in AGS cells with GFP-LC3B reporter signals by if assays was analyzed by ImageJ. Data are presented as the mean ± SD of six independent images, **p < .01. (d) The representative images illustrate mCherry- GFP-LC3 reporter signals by if assays in *H. pylori*-infected (Hp)or un-infected (UI) AGS cells, as well as *H. pylori*-infected AGS with control or AUF1 siRNA. The experiment was independently repeated three times. Scale bar 10 μm. Immunofluorescence intensity of red dots (RD) and green dots (GD) in cell was analyzed by ImageJ. Data are presented as the mean ± SD of six independent images, ***p < .001. (e) Ultrastructural features in *H. pylori* -infected (HP) or un-infected (UI) AGS cells, as well as *H. pylori*-infected AGS with control or AUF1 siRNA were analyzed by transmission electron microscopy. Scale bar 2 µm (left). The experiment was independently repeated three times. The typical images of autophagosomes (AP, one arrow) and autolysosomes (AL, two arrows) are shown at higher magnification. Data are presented as the mean ± SD of 10 independent images, **p < .01.

To investigate the role of AUF1 on autophagy in gastric epithelial cells, we detected the levels of LC3B and p62 in AGS, GES-1 cells and human primary gastric epithelial cells by Western blot assays. The conversion of LC3B-I to LC3B-II is one of the hallmarks of autophagosome formation, whereas p62 degradation indicates a dynamic autophagic flux.^24^ We observed that AUF1-knockdown cells exhibited increased levels of LC3B-II and decreased levels of p62 (Figure 1(a)). Conversely, when we overexpressed the four separate isoforms of AUF1, we observed the accumulation of both LC3B-II and p62 proteins, indicating that AUF1 plays a role in blocking autophagic flux (Figure S1a).

It has also been reported that *H. pylori* infection resulted in the accumulation of LC3B-II and p62,^14,25,26^ as also confirmed in Figure 1(b), indicating a blockage of autophagic flux. We also observed upregulation of AUF1 after *H. pylori* infection, as previously found^23^ (Figure 1(b)). To further investigate the effect of AUF1 on autophagy during *H. pylori* infection, we conducted AUF1-knockdown experiments in AGS, GES-1 cells and human primary gastric epithelial cells infected with *H. pylori*. We observed an increase in LC3B-II protein levels and a decrease in p62 levels upon AUF1-knockdown treatment in *H. pylori*-infected cells, suggesting that AUF1 knockdown can restore the impaired autophagic flux caused by *H. pylori* infection (Figure 1(b)). These findings were further supported by our observations in the GFP-LC3 reporter system, where *H. pylori* infection caused autophagosomes accumulation, and both AUF1-knockdown and overexpressed cells exhibited more autophagosomes accumulation (Figure 1c & S1b). Furthermore, in the GFP-mCherry-LC3 reporter system, we noticed an increased presence of mCherry-LC3 signals compared to GFP-LC3 signals in *H. pylori*-infected cells with AUF1-knockdown, while the opposite was observed in *H. pylori*-infected cells with AUF1-overexpression (Figure 1(d) & S1c), suggesting AUF1-knockdown can eliminate blockage of autophagic flux by *H. pylori* infection, and AUF1 overexpression further aggravates the blockage by *H. pylori* infection. Finally, transmission electron microscopy analysis revealed that *H. pylori* infection induced autophagosome formation, while a higher number of autolysosomes (AL) than autophagosomes (AP) in AUF1-knockdown cells compared to control cells (Figure 1(e)). These results collectively indicate that AUF1 acts as an inhibitor of autophagic flux during *H. pylori* infection and *H. pylori* inhibits autophagic flux by upregulating AUF1 expression.

### AUF1 stabilizes CagA protein levels by inhibiting autolysosomal degradation of intracellular CagA

**Figure 2.**
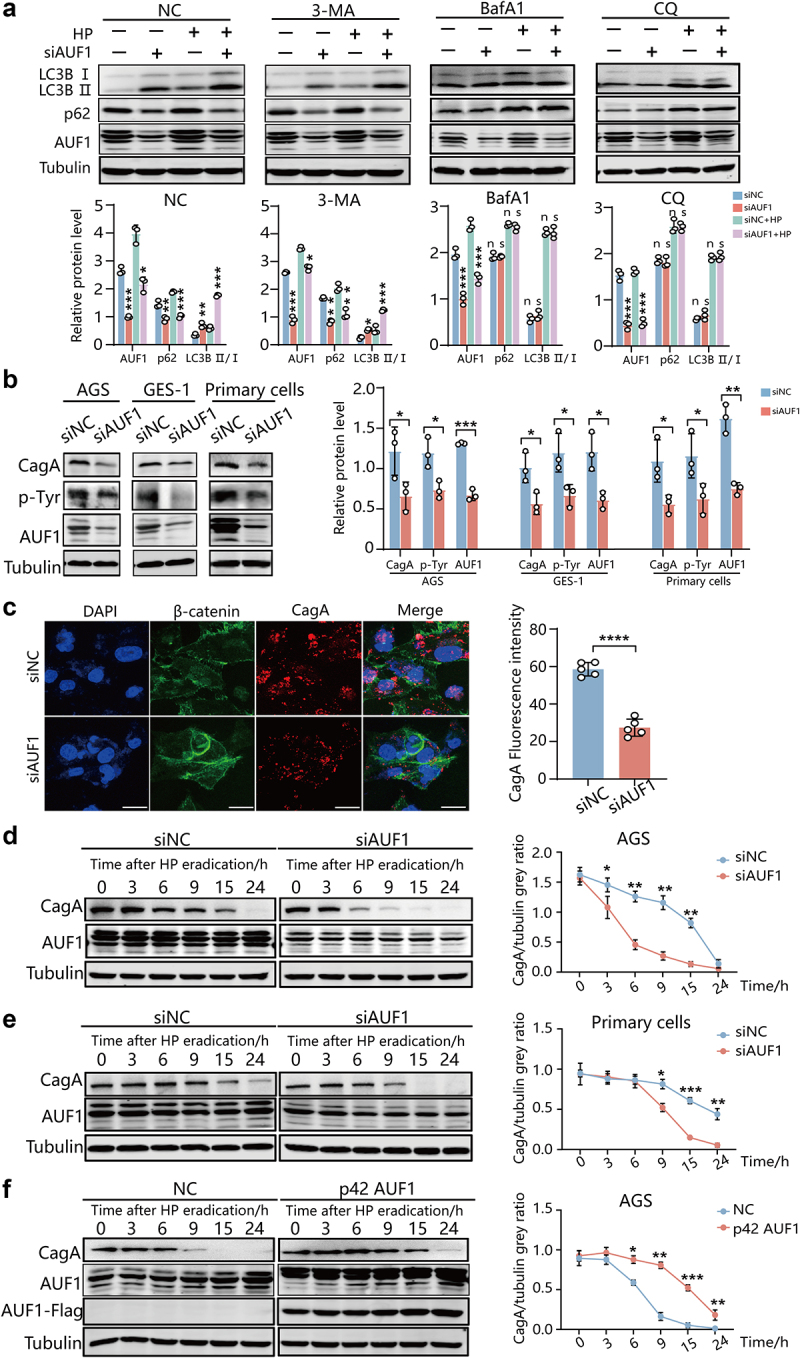
AUF1 inhibits autolysosomal degradation of intracellular CagA. (a) After treatment with 10 mM 3-MA, 50 μM CQ or 10 μM Baf A1, LC3B, p62 and AUF1 expression was analyzed by Western blot assays in AGS cells with control or AUF1 siRNA during un-infection or *H. pylori* infection. Gray values of LC3B, p62 and AUF1 band were analyzed by ImageJ. LC3B, p62 and AUF1/tubulin gray ratio was quantified, all the quantitative data are presented as means ± SD from three independent experiments. *p < .05, **p < .01, ***p < .001, ns means not significant. (b) CagA, *p*-tyr and AUF1 expression was analyzed by Western blot assays in *H. pylori* -infected AGS cells, GES-1 cells and human primary gastric epithelial cells after AUF1 knockdown or not. Gray values of CagA, *p*-tyr and AUF1 band were analyzed by ImageJ. CagA, *p*-tyr and AUF1/tubulin gray ratio was quantified, all the quantitative data are presented as means ± SD from three independent experiments. *p < .05, **p < .01, ***p < .001. (c) Utilizing β-catenin as apical and basal marker, CagA expression was analyzed by if assays in *H. pylori* -infected GES-1 cells after AUF1 knockdown or not. The experiment was independently repeated three times. Scale bar 25 μm. Immunofluorescence intensity of red in cell was analyzed by ImageJ. Data are presented as the mean ± SD of five independent images. ****p < .0001. (d–f). After 5 h of *H. pylori* 26695 infection, the AGS cells (d) and human primary gastric epithelial cells (e) with AUF1 knockdown or the AGS cells with p42-AUF1 overexpression (f) were incubated with gentamicin for 1 h to kill extracellular bacteria. Subsequently, CagA expression at indicated times was analyzed by Western blot assays. Gray values of CagA band were analyzed by ImageJ and CagA/tubulin gray ratio was quantified, all the quantitative data are presented as means ± SD from three independent experiments. *p < .05, *p* < .01, ***p < .001.

To determine whether AUF1 affects autophagy induction (autophagosome formation) or lysosomal clearance, we evaluated LC3B-II and p62 levels in the presence of inhibitor of autophagy induction 3-methyladenine (3-MA), which is a PtdIns3K inhibitor that effectively blocks an early stage of autophagy,^24^ and lysosome inhibitors bafilomycin A1 (BafA1) and Chloroquine (CQ). Both BafA1 and CQ could cause alkalinization of the lumen and thus impair lysosomal function, although with distinct mechanisms. BafA1 selectively inhibits the vacuolar-type H^+^-ATPase, preventing lysosomal acidification and autophagosome-lysosome fusion^27^ while CQ primarily inhibits lysosomal acidification.^28^ Our data showed that treatment with 3-MA effectively decreased the levels of LC3-II and p62 in both AUF1-knockdown cells and control cells. However, the regulatory effects of AUF1 knockdown on LC3B-II and p62 still persisted (Figure 2(a)), indicating that AUF1 does not affect the initiation of autophagy. Additionally, when we treated the cells with CQ or BafA1, AUF1 knockdown did not further increase LC3B II levels or reduce p62 levels compared to control cells (Figure 2(a)). Similarly, the stimulatory effect of AUF1 overexpression on LC3B II and p62 was abolished under BafA1 treatment (Figure S2). These findings confirm that AUF1 impairs lysosomal clearance rather than affecting autophagosome formation.

To investigate AUF1’s role on autolysosomal degradation of intracellular CagA, we conducted knockdown and overexpression experiments of AUF1 in an in vitro *H. pylori* infection model. The results showed that the level of CagA and phosphorylated-tyrosine (p-Tyr) decreased upon AUF1 knockdown, while it increased after overexpressing different AUF1 isoforms (Figure 2(b,c), S3). These findings suggest that AUF1 may enhance the stability of intracellular CagA. To further explore the effect of AUF1 on CagA stability, we incubated AGS cells with gentamicin (Gen) to kill extracellular bacteria after 5 h post *H. pylori* infection and perform CagA protein turnover assays. The results showed that the half-life of intracellular CagA decreased from 15 to 5 h in *H. pylori-*infected AGS cells and from 20 to 9 h in primary gastric epithelial cells upon AUF1 knockdown, respectively (Figure 2(d,e)). Moreover, the degradation rate of p-Tyr was also accelerated in *H. pylori*-infected AGS and primary gastric epithelial cells with AUF1 knockdown (Figure S4). Similarly, in *H. pylori*-infected GES-1 cells with reduced AUF1 expression, the half-life of intracellular CagA decreased from 9 to 5 h (Figure S5a). Conversely, overexpression of p42 AUF1 extended the half-life of intracellular CagA (Figure 2(f)).

Next, we examined whether AUF1 also affects the stability of exogenously overexpressed CagA protein. The results showed that the levels of exogenous CagA in AUF1-knockdown cells were significantly lower than those in control cells in a time-dependent manner (Figure S5b,c). On the contrary, overexpression of p42 AUF1 extended the half-life of exogenous CagA in CagA-overexpressed AGS cells (Figure S5d). These findings demonstrated that AUF1 enhances CagA protein stability by inhibiting CagA degradation. Furthermore, we utilized BafA1 and CQ to inhibit autolysosomal degradation in *H. pylori*-infected AGS cells. By inhibiting the autolysosomal degradation process, we observed that the effect of AUF1 knockdown in promoting CagA degradation was abolished (Figure S6a,b). This suggests that AUF1 stabilizes CagA levels by inhibiting autolysosomal degradation.

### Transcriptome analysis reveals that AUF1 inhibits autolysosomal degradation by regulating the expression of lysosomal-associated hydrolase genes

**Figure 3.**
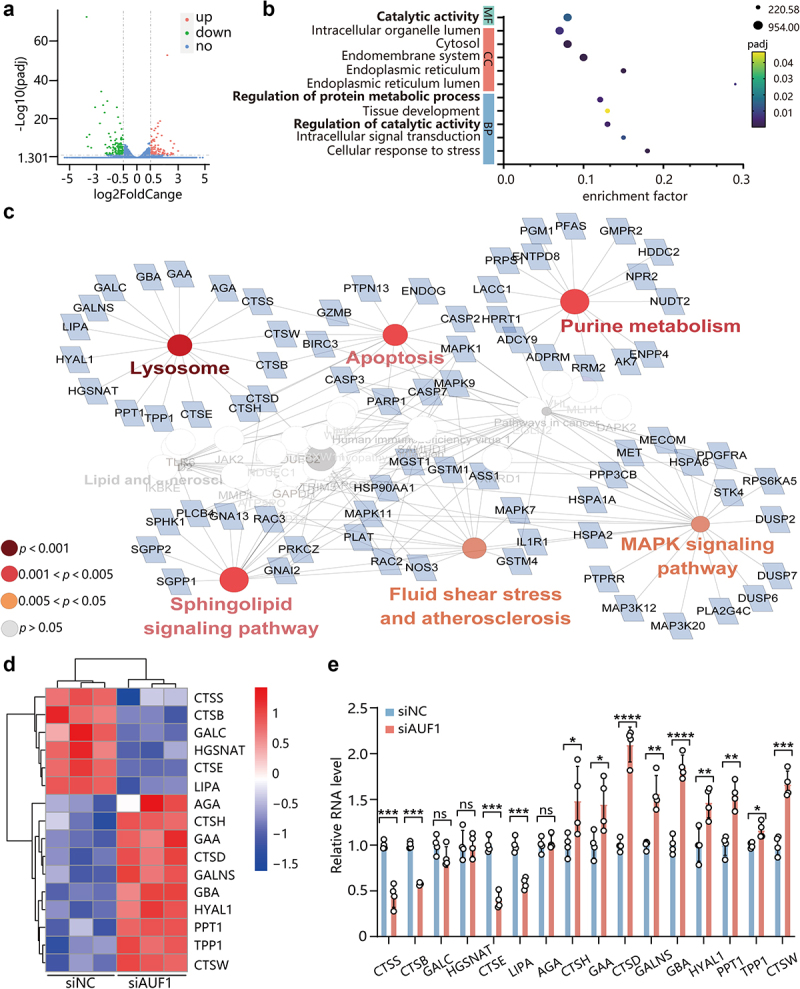
Transcriptome analysis shows that AUF1 inhibits autolysosomal degradation by regulating the expression of lysosomal-associated hydrolase genes. (a) Differentially expressed genes in transcriptomics data of *H. pylori*-infected AGS cells with AUF1 knockdown. (b) GO analysis in transcriptomics data of *H. pylori*-infected AGS cells with AUF1 knockdown. (c) The top 10 enrichment pathways of KEGG pathway analysis based on GO analysis of 557 “catalytic activity”-related genes in molecular function. (d) Heat map analysis on 16 highly enriched lysosomal hydrolases genes among lysosome pathway. (e) Identified 16 highly enriched lysosomal hydrolases genes mRNA were analyzed by RT-qPCR in AGS cells with AUF1 knockdown during *H. pylori* infection. Data are expressed as means ± SD from three independent experiments. *p < .01, **p < .01, *p* < .001, ****p < .0001, ns means not significant.

To further explore the underlying mechanism by which AUF1 inhibits autolysosomal degradation, we conducted a transcriptome analysis in *H. pylori*-infected AGS cells with AUF1 knockdown. A total of 1996 differentially expressed genes were identified after knocking down AUF1, including 1029 upregulated genes and 967 downregulated genes (Figure 3(a)). Gene Ontology (GO) analysis showed that these differential genes were enriched in molecular functions and biological processes related to protein degradation, such as catalytic activity, regulation of protein metabolic process, and regulation of catalytic activity (Figure 3(b)).

Next, we performed KEGG pathway analysis on a set of 557 genes associated with “catalytic activity” in the molecular function category of the GO database. The analysis revealed that the top 10 enrichment pathways were lysosome, purine metabolism, Sphingolipid signaling pathway, apoptosis, MAPK signaling pathway, and so on (Figure 3(c) & Table S1). Within the lysosome pathway, we observed a significant enrichment of differentially expressed genes related to lysosomal hydrolases, including 16 lysosomal hydrolases such as CTSD, CSTB, CTSS and others (Figure 3(c) & Table S1). These enzymes are primarily involved in lysosomal degradation function. Notably, our transcriptome analysis following AUF1 knockdown demonstrated that *CTSS*, *CTSB*, *GALC*, *HGSNAT*, *CTSE* and *LIPA* were downregulated, while *AGA*, *CTSH*, *GAA*, *CTSD*, *GALNS*, *GBA*, *HYAL1*, *PPT1*, *TPP1* and *CTSW* were upregulated (Figure 3(d)). The expression of these 16 genes after AUF1 knockdown was further verified by RT-qPCR. Among these genes, *CTSD* exhibited the most significant upregulation by *AUF1* depletion, followed by *GBA*, *CTSW*, *CTSH*, *GALNS*, etc. (Figure 3(e)). Taken together, these results suggest that AUF1 may affect autolysosomal degradation by regulating the expression of lysosomal-associated hydrolase genes.

The limitation of autolysosomal degradation may be due to the blocked fusion of autophagosome and lysosome, suboptimal lysosomal pH, insufficient lysosomal enzymes and Ca^2+^ release.^29–31^ To further investigate the effects of AUF1 on lysosome function, we conducted several experiments to test the fusion of autophagosome and lysosome, lysosomal acidification and Ca^2+^ release. Firstly, we utilized the GFP-LC3 reporter system to label autophagosomes and observed that the colocalization of GFP-LC3 with the late endosomal lysosomal marker LAMP1^32^ increased with AUF1 depletion, regardless of *H. pylori* infection. This suggests that AUF1 knockdown-induced autophagosomes acquired the late endosomal lysosomal marker LAMP1, indicating that autophagosome-lysosome fusion was not affected (Figure S7). Next, we assessed lysosomal pH using LysoTracker Red staining and LysoSensor Yellow/Blue DND-160 (PDMPO) assays.^33^ Interestingly, regardless of *H. pylori* infection, AUF1 does not appear to play a role in lysosomal acidity (Figure S8a,b). Additionally, we found that knockdown of AUF1 did not impact Ca^2+^ release or the expression of key lysosomal degradation-related genes (Figure S9a,b).

### AUF1 binds to the 3’UTR region of CTSD mRNA and downregulates CTSD mRNA expression

**Figure 4.**
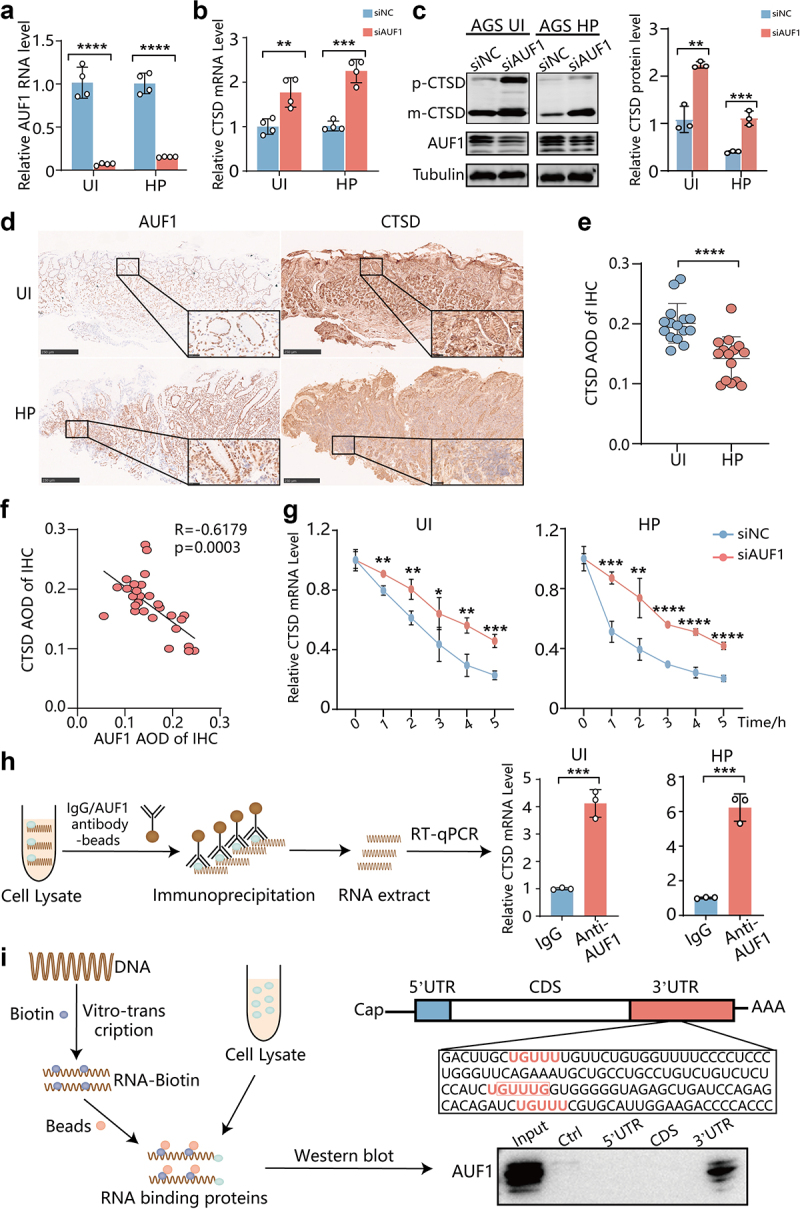
AUF1 inhibited the expression of CTSD by targeting the 3’UTR region of CTSD mRNA.

It has been reported that CTSD is a cathepsin which is significantly lacked in gastric epithelial cells with persistent *H. pylori* infection, thus disrupting the autophagic pathway and resulting in a failure of the autophagic degradation function.^34^ Interestingly, our results also identified CTSD as one of the lysosomal-associated protease genes that were most significantly upregulated (Figure 3(e)). Therefore, we focused on investigating the regulatory role of AUF1 on CTSD. We observed that CTSD expression was upregulated upon AUF1 knockdown in both uninfected and *H. pylori*-infected AGS cells (Figure 4(a-c)). Additionally, immunofluorescence assay showed reduced CTSD levels in *H. pylori*-infected AGS cells with AUF1 overexpression (Figure S10a). Notably, neither the autophagy activator Rapamycin (Rapa) nor autophagy inhibitor BafA1 had any effect on the regulation of CTSD mRNA by AUF1 (Figure S10b,c). Immunohistochemistry (IHC) assay showed that the expression of CTSD was significantly decreased in *H. pylori*-infected gastric mucosa tissues of patients with superficial gastritis, along with upregulation of AUF1 (Figure 4(d,e)). These findings suggest a negative correlation between the expression level of AUF1 and CTSD in these clinical patients (Figure 4(f)).

(a) AUF1 mRNA was analyzed by RT-qPCR in AGS cells with AUF1 knockdown during un- infection or *H. pylori* infection. (b) CTSD mRNA was analyzed by RT-qPCR in AGS cells with AUF1 knockdown during un-infection or *H. pylori* infection. **p < .01, ****p < .0001, ****p < .0001. (c) CTSD protein was analyzed by Western blot assays in AGS cells with AUF1 knockdown during un-infection or *H. pylori* infection. Gray values of CTSD band were analyzed by ImageJ. CTSD/tubulin gray ratio was quantified, all the quantitative data are presented as means ± SD from three independent experiments. **p < .01, ***p < .001. (d) Representative immunohistochemistry images of CTSD and AUF1 from gastric mucosa of clinical superficial gastritis patients with un-infection or *H. pylori* infection. Scale bar 250 μm. (e) Average density of CTSD in gastric mucosa of 30 clinical superficial gastritis patients with or without *H. pylori* infection by IHC (*n* = 15, per group). Data is expressed as means ±SD. ****p < .0001. (f) Correlation analysis of average density of AUF1 and CTSD in gastric mucosa of clinical superficial gastritis patients with un-infection or *H. pylori* infection. (g) The stability of CTSD mRNA was measured in AGS cells with AUF1 knockdown during un-infection or *H. pylori* infection following incubation with actinomycin D for the times indicated. The levels of CTSD mRNA were assayed by RT-qPCR. Data are presented as the mean ± SD of three independent tests. *p < .05, **p < .01. (h) The flow diagram illustrating the experimental procedure of RIP assays. CTSD mRNA level pulled-down by AUF1 in AGS cells during un-infection or *H. pylori* infection were assayed by RT- qPCR. Data are expressed as means ± SD of three independent tests, ***p < .001. (I) The flow diagram illustrating the experimental procedure of RNA-pulldown assays. The schematic diagram of the full‐length human CTSD mRNA was shown and the mRNA sequence of 3’UTR (142–279 bp) of CTSD mRNA was listed in the lower box. Biotinylated RNA segments of CTSD mRNA were used to pulldown lysates of AGS cells, and the pulldown materials were analyzed by Western blot assay.

We then proceeded to investigate the mechanism by which AUF1 regulates CTSD expression. Through mRNA turnover assays, we found that knockdown of AUF1 increased the half-life of CTSD mRNA from 3 h to 5 h in uninfected AGS cells, and from 1 to 5 h in *H. pylori*-infected AGS cells (Figure 4(g)), suggesting AUF1 destabilized CTSD mRNA. Furthermore, we examined the binding of AUF1 to CTSD mRNA in cells using RNA immunoprecipitation (RIP) assay (Figure 4(h)). The results revealed a higher enrichment of CTSD mRNA in the precipitates obtained with anti-AUF1 antibody compared to the IgG control, indicating that AUF1 can indeed bind to CTSD mRNA (Figure 4(h) & S11). To further investigate the specific binding sites of AUF1 on CTSD mRNA, we analyzed the sequence of the 3’UTR region and identified four nonclassical GU- or UG-rich sequences (UGUUU and GUUUG) that have been previously identified as potential binding sites for AUF1^19^ (Figure 4(i)). To confirm this interaction, we performed RNA-pulldown assays using in vitro-synthesized biotinylated CTSD RNAs. The results showed that AUF1 preferentially bound to the 3’UTR region of CTSD mRNA compared to the coding sequence (CDS) and 5’UTR regions (Figure 4(i)). Taken together, these findings provide evidence that AUF1 binds to the 3’UTR region of CTSD mRNA and downregulates CTSD expression.

### AUF1-mediated downregulation of CTSD expression contributes to CagA stability

**Figure 5.**
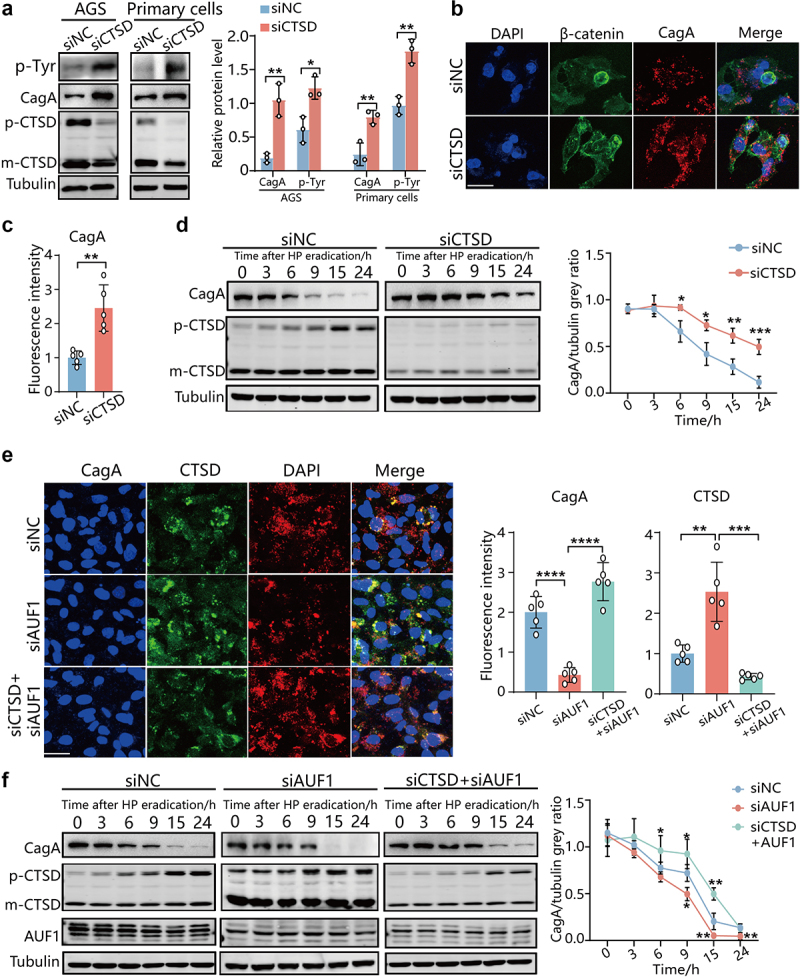
AUF1 inhibits CagA degradation by inhibiting CTSD expression. (a) CagA and *p*-Tyr levels were analyzed by Western blot assays in AGS cells and human primary gastric epithelial cells with CTSD knockdown during *H. pylori* infection. Gray values of CagA and *p*-Tyr band were analyzed by ImageJ. CagA and *p*-Tyr/tubulin gray ratio was quantified, all the quantitative data are presented as means ± SD from three independent experiments. *p < .05, **p < .01. (b) Utilizing β-catenin as apical and basal marker, intracellular CagA levels were analyzed by if assay in *H. pylori*-infected GES-1 cells with CTSD knockdown. The experiment was independently repeated three times. Scale bar 25 μm. (c) Immunofluorescence intensity of red in cell was analyzed by ImageJ. Data are presented as the mean ± SD of five independent images. **p < .01. (d) After *H. pylori* 26695 infected for 5 h, the AGS cells with CTSD knockdown were incubated with gentamicin for 1 h to kill extracellular bacteria. Subsequently, CagA expression at indicated times was analyzed by Western blot assays. Gray values of CagA band were analyzed by ImageJ and CagA/tubulin gray ratio was quantified, all the quantitative data are presented as means ± SD from three independent experiments. *p* < .05, **p < .01, ***p < .001. (e) CTSD and CagA levels were analyzed by IF assay in *H. pylori*-infected human primary gastric epithelial cells with control, AUF1 knockdown, AUF1 and CTSD knockdown. The experiment was independently repeated three times. Scale bar 25 μm. Immunofluorescence intensity of red and green in cell was analyzed by ImageJ. Data are presented as the mean ± SD of five independent images. **p < .01, ***p < .001, ****p < .0001. (f) After *H. pylori* 26695 infected for 5 h, the AGS cells with control, AUF1 knockdown, AUF1 and CTSD knockdown were incubated with gentamicin for 1 h to kill extracellular bacteria. Subsequently, CagA expression at indicated times was analyzed by Western blot assays, Gray values of CagA band were analyzed by ImageJ and CagA/tubulin gray ratio was quantified, all the quantitative data are presented as means ± SD from three independent experiments. *p < .05, **p < .01, ***p < .001.

To investigate whether AUF1 regulates CagA degradation by downregulating CTSD expression, we first tested the effects of CTSD on intracellular CagA in *H. pylori*-infected AGS cells. The Western blot and immunofluorescence assays revealed that knockdown of CTSD significantly increased CagA and p-Tyr levels (Figure 5(a-c)). Consistently, the levels of intracellular CagA in CTSD-knockdown cells were significantly higher than those in control cells over time (Figure 5(d)), suggesting that CTSD knockdown prolonged the half-life of intracellular CagA in *H. pylori-*infected AGS cells. These results suggest that CTSD knockdown enhanced CagA protein stability.

Additionally, we investigated the impact of AUF1 on CagA levels and protein stability by regulating CTSD expression. Knockdown of AUF1 resulted in elevated CTSD expression and decreased CagA accumulation. However, simultaneous knockdown of CTSD abolished the promoting effect of CagA degradation by AUF1 knockdown (Figure 5(e)). Similarly, AUF1 knockdown shortened the half-life of CagA and was accompanied by significant upregulation of CTSD, while simultaneous knockdown of CTSD counteracted the promoting effect of CagA protein degradation caused by AUF1 knockdown (Figure 5(f)). These results collectively indicate that AUF1 inhibits CagA degradation by suppressing CTSD expression.

### AUF1 promotes secretion of CagA in exosomes and induces extracellular inflammatory cytokine expression

It has been reported that *H. pylori*-infected gastric epithelial cells can secrete exosomes containing CagA, which contribute to the development of extragastric disorders.^15,16^ Inhibiting lysosomal function or autophagy can promote exosome secretion.^35,36^ However, it remains unclear whether AUF1 can affect the secretion of CagA via exosomes. To investigate this, we isolated exosomes from the supernatant of *H. pylori*-uninfected and *H. pylori*-infected AGS cells using ultracentrifugation. Transmission electron microscopy (TEM) and nanoparticle tracking analysis (NTA) confirmed that these exosomes primarily consisted of double-layered vesicles with a diameter distribution ranging from 110 to 150 nm (Figure S12a,b). Consistent with previous reports, CagA was also detected in exosomes derived from wild-type *H. pylori* (WT-HP) (Figure S12c).

**Figure 6.**
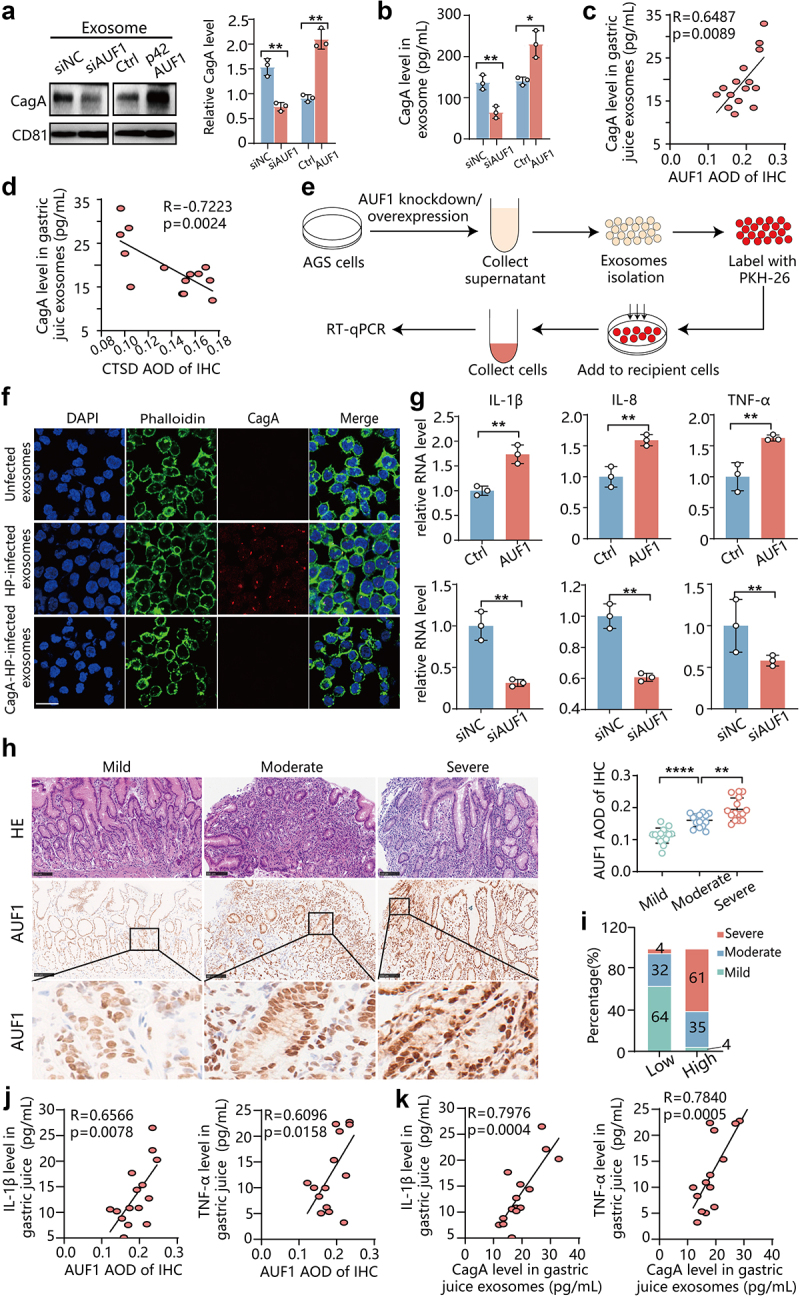
AUF1 promoted secretion of CagA in exosomes and upregulate extracellular inflammatory cytokine expression. (a & b) CagA expression was analyzed by Western blot assays (a) and Elisa assays (b) in exosomes of AGS cells supernatant during *H. pylori* infection with AUF1 knockdown or AUF1 expression. Data of Western blot and Elisa assays are presented as the mean ± SD of three independent tests. *p < .05, **p < .01. (c & d) CagA expression was analyzed by Elisa assays in gastric juice exosomes of 15 clinical superficial gastritis patients with *H. pylori* infection. Correlation analysis between CagA expression in gastric juice exosomes and AOD of AUF1(c) or AOD of CTSD (d) by IHC in gastric mucosa of same clinical superficial gastritis patients with *H. pylori* infection. (e) The flow diagram illustrating the incubation process of AGS exosomes derived from *H. pylori*-infected cells with recipient cells. (f) AGS cells were infected with wild-type or CagA-knockout *H. pylori* or uninfected for 48 h, then the cell supernatant was collected and purified to harvest exosomes by ultracentrifugation. The AGS -derived exosomes were incubated with GES-1 cells and CagA expression of GES-1 cells was analyzed by IF assay. Scale bar 25 μm. (g) IL-1β, IL-8 and TNF-α mRNA level in GES-1 cells incubating with exosomes from *H. pylori*-infected AGS cells with AUF1 overexpression or AUF1 knockdown. Data are expressed as means ± SD of three independent tests. **p < .01. (h) The representative images illustrate the immunohistochemical expression of AUF1 in the gastric tissues of individuals from *H. pylori*-infected chronic superficial gastritis with different degrees of inflammation, including mild, moderate and severe (*n* = 15, per group). Scale bar 100 μm. AOD of AUF1 in total cell of immunohistochemistry was analyzed by ImageJ in the gastric tissues of these individuals. Data are expressed as means ± SD. **p < .01, ****p < .0001. (i) Percentage of mild, moderate, and severe *H. pylori*-infected chronic superficial gastritis in low AUF1 expression group and high AUF1 expression group. (j) Correlation analysis between AOD of AUF1 by IHC in gastric mucosa and IL-1β/TNFα expression in gastric juice of same clinical superficial gastritis patients with *H. pylori* infection. (k) Correlation analysis between CagA expression and IL-1β/TNFα expression in gastric juice of same clinical superficial gastritis patients with *H. pylori* infection.

To explore the impact of AUF1 on CagA secretion, we isolated exosomes from *H. pylori*-infected AGS cells with either AUF1 knockdown or AUF1 overexpression. Surprisingly, we observed a significant reduction in CagA levels in exosomes derived from *H. pylori*-infected AGS cells with AUF1 knockdown, while overexpression of AUF1 led to a significant increase in CagA level in exosomes (Figure 6(a,b)). To further evaluate the effect of AUF1 on CagA in exosomes, we collected gastric juice from patients with and without *H. pylori* infection and measured the CagA levels in exosomes using an ELISA assay. The results revealed the levels of CagA in the gastric juice exosomes of *H. pylori*-infected patients positively correlated with AUF1 expression in their gastric mucosa (Figure 6(c)), while negatively correlating with CTSD expression (Figure 6(d)). Taken together, these results suggest that AUF1 may impair the degradation of CagA by downregulating CTSD expression, consequently leading to increased CagA secretion in exosomes.

It is well known that CagA can aggravate gastric mucosal inflammation via activating NF-κB pathways.^11^ To examine the role of exosome-mediated CagA on extracellular inflammation, we incubated these exosomes with GES-1 cells as illustrated in Figure 6(e). Staining with PKH26 dye demonstrated that exosomes derived from AGS cells were taken up by GES-1 cells in a time-dependent manner (Figure S13). After 48 h of incubation, CagA was detected in GES-1 cells, having entered via exosomes derived from WT-*H. pylori*-infected AGS cells. (Figure 6(f)). The exosomes from WT-*H. pylori*-infected AGS cells upregulated the mRNA levels of interleukin-1β (IL-1β), interleukin-8 (IL-8) and tumor necrosis factor-α (TNF-α) in GES-1 and RAW264.7 cells compared to exosomes from CagA-knockout-*H. pylori*-infected AGS cells (Figure S14a, b), suggesting CagA can promote the expression of extracellular inflammatory cytokines. Consistently, GES-1 cells treated with exosomes from *H. pylori*-infected AGS cells overexpressing AUF1 exhibited higher mRNA levels of IL-1β, IL-8, and TNF-α compared to cells treated with exosomes from control cells (Figure 6(g)). Furthermore, both GES-1 cells (Figure 6(g)) and Raw264.7 cells (Figure S14c) showed lower mRNA levels of IL-1β, IL-8, and TNF-α after treatment with exosomes from *H. pylori*-infected AGS cells with AUF1 knockdown. Taken together, these results suggest that AUF1 can upregulate the expression of extracellular inflammatory cytokines by promoting the secretion of CagA-containing exosomes.

Furthermore, we observed a gradual increase in AUF1 expression with the aggravation of inflammation in the gastric mucosal tissues of *H. pylori-*infected patients with chronic superficial gastritis by IHC analysis (Figure 6(h)). Notably, within the same gastric mucosa sample, there was a positive correlation between the severity of inflammation and the strength of AUF1 expression (Figure 6(h)). Next, we divided the gastric mucosa samples into low AUF1 expression group and high AUF1 expression group based on the median average optical density (AOD) value of AUF1 expression. We observed that mild superficial gastritis (64%) and moderate superficial gastritis (32%) were mainly found in the low AUF1 expression group, while moderate superficial gastritis (35%) and severe superficial gastritis (61%) were mainly found in the high AUF1 expression group (Figure 6(i)). Moreover, the expression levels of AUF1 in gastric mucosal tissues and CagA in gastric juice exosomes were positively associated with the levels of IL-1β and TNF-α within the gastric juice in *H. pylori-*infected patients (Figure 6(j,k)). These findings suggest that AUF1 is involved in *H. pylori*-associated gastritis and provide further support for its role in upregulating the expression of extracellular inflammatory cytokines.

### CagA is necessary to facilitate the translocation of AUF1 into the cytoplasm

**Figure 7.**
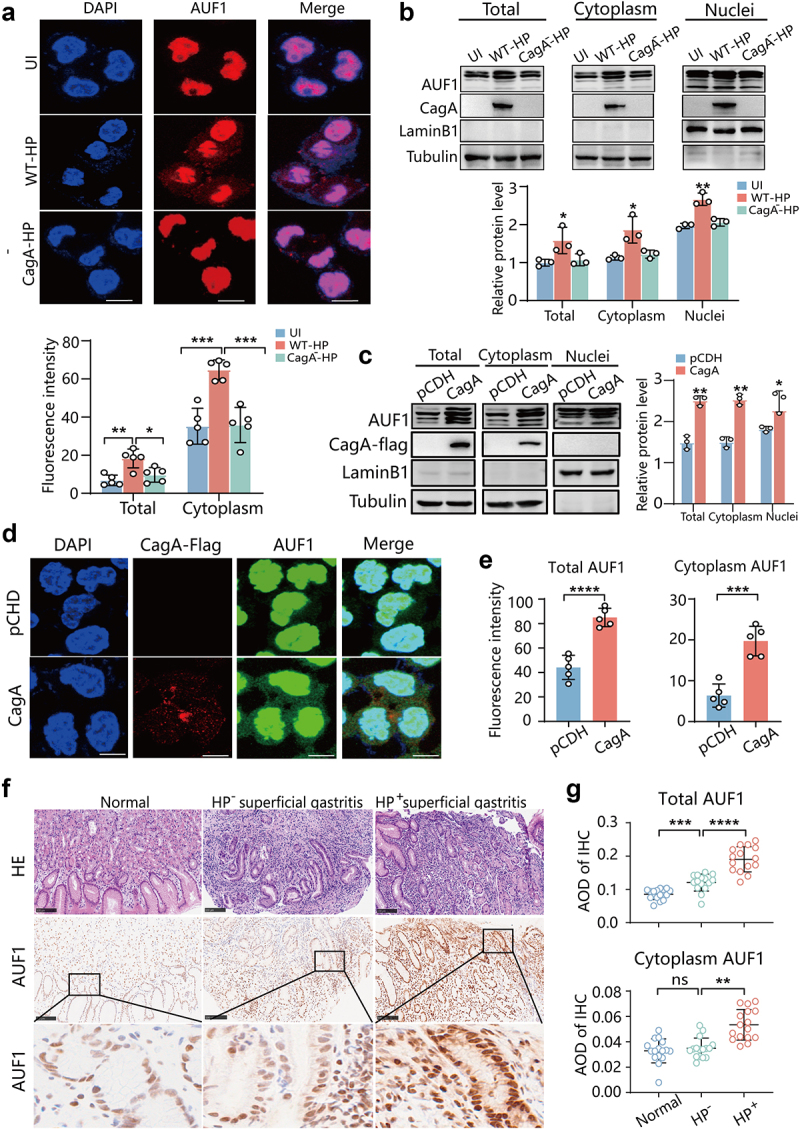
CagA increased AUF1 expression and cytoplasmic localization. (a&b). AUF1 expression and subcellular localization were analyzed by immunofluorescence assays (a) and Western blot assays (b) and after nuclear and cytoplasmic protein extraction of AGS cells with un- infection (UI) or wild *H. pylori* (WT-HP) or CagA-knockout *H. pylori* (CagA^−^-HP) infection for 24 h. The experiment was independently repeated three times. Immunofluorescence intensity of AUF1 in total cell and cytoplasm was analyzed by ImageJ in if assays. Data are presented as the mean ± SD of five independent images. Scale bar 10 μm. Gray values of AUF1 band were analyzed by ImageJ and AUF1/tubulin gray ratio was quantified. Data of Western blot assays are presented as the mean ± SD of three independent tests. *p < .05, **p < .01, ***p < .001. (c & d) AUF1 expression and subcellular localization were analyzed by nuclear and cytoplasmic protein extraction (c) and if assays (d) in AGS cells transfected with exogenous CagA-overexpressing plasmid. Gray values of AUF1 band were analyzed by ImageJ and AUF1/tubulin gray ratio was quantified. Data of Western blot assays are presented as the mean ± SD of three independent tests. Scale bar 8 μm. (e) Immunofluorescence intensity of AUF1 in total cell and cytoplasm was analyzed by ImageJ in if assays (d). Data are presented as the mean ± SD of five independent images. ***p < .001, *p* < .0001. (f) The representative images illustrate the immunohistochemical expression and cytoplasmic redistribution of AUF1 in the gastric tissues of humans, including normal individuals, individuals with- and without-*H.pylori* infection in chronic superficial gastritis (*n* = 15, per group). Scale bar 100 μm. (g) AOD of AUF1 in total cell and cytoplasm of immunohistochemistry was analyzed by ImageJ in the gastric tissues of individuals, individuals with- and without-*H.pylori* infection in chronic superficial gastritis. Data is expressed as means ± SD. **p < .01, ***p < .001, *p* < .0001, ns means not significant..

Typically, AUF1 is localized predominantly in the nuclei of cells. However, under certain stressful conditions, such as viral infection like coxsackievirus, enterovirus and HIV-1, AUF1 undergoes significant nuclear to cytoplasmic translocation to involve in viral genome stability and viral protein synthesis.^20,37–39^ Therefore, AUF1 plays a crucial role in the regulation of infection. Our previous studies have shown that the expression of AUF1 could be upregulated by CagA of *H. pylori* in GES-1 cells.^23^ It is intriguing to explore whether the cytoplasmic translocation of AUF1 is associated with CagA of *H. pylori*. To investigate this, we conducted cytoplasmic protein extraction and immunofluorescence assays in AGS cells infected with wild-type or CagA-knockout *H. pylori*, as well as in uninfected cells. As depicted in Figure 7(a,b), infection with wild-type *H. pylori* (WT-HP) not only upregulated the expression of AUF1 but also facilitated its translocation to the cytoplasm. Conversely, the CagA-knockout *H. pylori* (CagA^−^- HP) strain did not exert the same effect on AUF1 localization. To further confirm the role of CagA in AUF1 expression and subcellular localization, we constructed an exogenous CagA-overexpressing plasmid and transfected it into AGS cells. Western blot analysis showed a significant increase in AUF1 protein levels in CagA-overexpressing AGS cells (Figure S15). Moreover, cytoplasmic protein extraction and immunofluorescence assays revealed that exogenous CagA promoted the expression and cytoplasmic localization of AUF1 (Figure 7(c–e)). Collectively, these results demonstrate that CagA not only increases AUF1 expression but also facilitates its localization to the cytoplasm.

To assess the cytoplasmic translocation of AUF1 in *H. pylori*-associated gastritis, we initially conducted IHC analysis on the gastric mucosa of 45 individuals. This included 15 samples from healthy individuals, 15 samples from patients with *H. pylori*-positive superficial gastritis, and 15 samples from patients with *H. pylori*-negative superficial gastritis. The results revealed a significant increase in both the expression and cytoplasmic translocation of AUF1 in the gastric mucosa of *H. pylori*-infected patients compared to healthy controls and uninfected patients with chronic superficial gastritis (Figure 7(f)). Furthermore, quantitative analysis of the IHC results showed that the AOD of total and cytoplasmic AUF1 in the gastric mucosa of *H. pylori*-infected patients with chronic superficial gastritis was significantly higher than that in healthy participants and uninfected patients with chronic superficial gastritis (Figure 7(g)). Interestingly, we also observed elevated levels of AUF1 in the gastric mucosa of uninfected patients with chronic superficial gastritis compared to healthy participants (Figure 7(f,g)). Taken together, these results suggest that the expression and cytoplasmic translocation of AUF1 are associated with *H. pylori* infection.

## Discussion

The interaction between *H. pylori* and the host is extremely complex. On one hand, *H. pylori* colonizes the gastric mucosa and induces inflammation as a defense mechanism by utilizing various virulence factors, while the host aims to clear *H. pylori*. On the other hand, in order to establish a continuous colonization in the protective gastric environment, *H. pylori* relies on specific host factors to safeguard its own virulence factors and evade host defense mechanisms, thereby maintaining damage to the gastric mucosa. The subtle balance between host clearance of *H. pylori* and *H. pylori* colonization requires further investigation. In our study, we identified AUF1 as a host factor exploited by *H. pylori* to protect CagA from rapid clearance by inhibiting autolysosomal degradation.

The effect of AUF1 on autophagy has been reported in other cells, but not in gastric epithelial cells. In RAW 264.7 macrophages and primary peritoneal macrophages, AUF1 cooperates with ZFP36L1 to destabilize BCL-2 mRNA, thus suppressing BCL-2 expression and promoting autophagy.^22^ In breast cancer cells, AUF1 stabilizes DDIT4 mRNA through binding, resulting in the inhibition of the mTOR signaling pathway and the induction of autophagy.^21^ In our study, we found that AUF1 plays a role in blocking autophagic flux in gastric epithelial cells regardless of *H. pylori* infection. These findings diverge from the observations made in macrophages and breast cancer cells, implying that the effects of AUF1 on autophagy may be cell-specific.

As a vital and widely studied virulence factor of *H. pylori*, CagA is directly injected into host cells through the type IV secretion system (T4SS) by *H. pylori*.^40^ The expression level of CagA depends on the genetic sequence of *H. pylori* itself.^41^ However, CagA is subject to degradation through the autophagy pathway within the host cell.^13,14^ Consequently, the level of CagA that has already entered the host cell is determined by the stability of the CagA protein within the cell. Translocated CagA will undergo tyrosine phosphorylation at EPIYA-motifs.^9,10^ Currently, the market faces a shortage of antibodies capable of effectively detecting the phosphorylated EPIYA motif within CagA.^9^ Thus, we utilized phosphorylated tyrosine as a true marker of intracellular CagA to confirm the findings in vitro. Notably, the regulatory effects of AUF1 on CagA and p-Tyr are consistent. Moreover, confocal microscopy utilizing β-catenin as apical and basal marker confirmed that CagA was indeed localized intracellularly.

We found that AUF1 acted downstream of autophagy pathway and impaired lysosomal clearance, rather than affecting autophagosome formation. Through manipulating the expression of AUF1, we demonstrated that AUF1 promotes the accumulation of CagA in gastric epithelial cells by inhibiting its autophagolysosomal degradation. Vacuolating cytotoxin A (VacA), another key virulence factor of *H. pylori*, also undergoes intracellular degradation via the autolysosomal pathway.^42^ Similarly, we observed a reduction in VacA protein levels after AUF1 knockdown (Fig S16), suggesting that AUF1 may inhibit the autophagolysosomal degradation of VacA. These findings suggest that AUF1 may have a broad inhibitory effect on various proteins through the autophagic lysosomal pathway, rather than specifically targeting the degradation of CagA.

Lysosomal hydrolases play a crucial role in lysosome function, as their expression and activity determine the degradation capacity of lysosomes.^29,43–45^ In our study, transcriptome analysis demonstrated that AUF1 affects the expression of various lysosomal-associated hydrolase genes, among which CTSD was the most significant. As an important proteolytic enzyme of the lysosome, CTSD is synthesized as an inactive precursor (53 kDa) in the rough endoplasmic reticulum, then converted into an active single-chain intermediate (48 kDa) in the endosome, and finally matures into a double-chain form (26 kDa) in the lysosomes.^46,47^ The function in enzymatic degradation of CTSD was positively correlated with lysosome acidity. Although BafA1 could not affect the regulation of CTSD mRNA by AUF1, it could reduce the catalytic activity of CTSD by changing the lysosomal pH. Thus, BafA1 and CQ could eliminate the pro-degradation effect of knockdown AUF1-mediated up-regulation of CTSD (Figure 2(a), S6a,b). Given that AUF1 regulates CTSD expression at the transcriptional level, it is logical to assume that AUF1 can impact both the pro-CTSD and mature CTSD levels. The canonical binding sequences of AUF1 are AU-rich sequences (AUUUA) found in mRNA 3’-UTR.^48–51^ Although the 3’UTR region of CTSD mRNA does not contain expected AU-rich sequences (AUUUA), it does contain four non-canonical GU- or UG-rich sequences (three UGUUU and one GUUUG),^19^ which may contribute to AUF1’s binding.

Interestingly, we also found that the expression of 16 lysosomal-associated hydrolase genes was generally downregulated in the presence of *H. pylori* infection alone (Figure S17), suggesting a compromised degradation capability within the gastric epithelial cells infected by *H. pylori*. Furthermore, it is noteworthy that among these downregulated genes, including *CTSH*, *GAA*, *CTSD*, *GALNS*, *GBA*, *PPT1*, *TPP1*, *CTSW*, etc., which are upregulated in *H. pylori*-infected AGS cells with AUF1 knockdown (Figure 3(e)), suggesting that down-regulation of these hydrolases following *H. pylori* infection may be mediated by AUF1, and CTSD is also the most significantly downregulated hydrolase mediated by AUF1 after *H. pylori* infection. However, other downregulated hydrolases following only *H. pylori* infection, such as CTSS, CTSB, CTSE, LIPA, etc., are also downregulated after AUF1 knockdown, which is inconsistent with the regulatory effect of AUF1, suggesting that these enzymes may be regulated by other host factors during *H. pylori* infection. Further investigation is needed to explore this mechanism in the future.

In line with previous researches^15,16^，we also observed the presence of CagA in the exosomes derived from *H. pylori*-infected cells. Inhibition of lysosomal function or autophagy has been shown to enhance exosome secretion.^35,36^ Consistently, we observed a decreased CagA level in exosomes from *H. pylori*-infected cells after AUF1 knockdown, while overexpression of p42 AUF1 increased CagA level in exosomes. This suggests that AUF1 promotes CagA secretion by inhibiting CagA degradation. However, the specific mechanism by which AUF1 affects CagA secretion, such as whether accumulated CagA is released more when its degradation is suppressed by AUF1 or if AUF1 itself directly promotes CagA secretion, requires further investigation. Consistent with previous reports,^52,53^ the ultrastructural features of exosomes purified from gastric juices were similar to those of exosomes isolated from AGS cells by TEM (Fig S12a, d). Although we were able to detect CagA in gastric juice using an ELISA assay in the presence of *H. pylori* infection, it was not detectable by western blot assay (data not shown), indicating that CagA levels in gastric juice are very low and may require more sensitive methods for detection.

Clinico-pathological observations strongly indicated proinflammatory actions of the CagA protein in gastric pathogenesis and CagA contributes to a pro-inflammatory microenvironment associated with the development of chronic gastritis through the activation of NF-κB pathway.^11,54^ In line with this, we also noted a positive correlation between the intensity of AUF1 expression, CagA level in gastric juices exosomes and the severity of inflammation in *H. pylori*-infected gastric mucosal tissues from patients (Figure 6). Moreover, the delivery of CagA by exosomes can also cause the upregulation of pro-inflammatory cytokines in recipient cells, which also showed a positive correlation with the expression of AUF1 in donor cells. Interestingly, we did not observe significant changes in the inflammation response pathway following AUF1 knockdown in gastric epithelial cells without *H. pylori* infection (Fig S18), suggesting that the positive correlation between AUF1 and inflammation may be mediated by CagA during *H. pylori* infection. These findings appear to contrast with previous observations in AUF1 knockout mice, where the knockout of AUF1 in mice was associated with the overexpression of TNFα and IL1β, culminating in severe symptoms of endotoxic shock.^55^ The complexity of microenvironment in vivo, the species difference of mice and humans, and the tissue heterogeneity may explain the different effects of AUF1 on inflammation regulation in knockout mice and in *H. pylori*-infected human gastric mucosal tissues and gastric epithelial cells. Further investigation is needed to explore this mechanism in the future.

Consistent with previous findings, our study demonstrated an increase in AUF1 expression in both gastric epithelial cells and gastric mucosal tissue of patients with chronic superficial gastritis infected with CagA-positive *H. pylori* (Figure 7). Although we did not conduct a biopsy typing test for CagA in the gastric mucosa of *H. pylori*-infected patients, recent studies in China have reported a prevalence of CagA-positive *H. pylori* strains ranging from 97% to 100%,^56,57^ suggesting that the majority of *H. pylori* strains infecting our study participants were CagA-positive. Furthermore, we found that both exogenous CagA and CagA-positive *H. pylori* infection can induce the translocation of AUF1 from the nucleus to the cytoplasm. Normally, AUF1 is predominantly located in the nucleus, but under certain stressful conditions, such as viral infection, it can be relocated to the cytoplasm to exert regulatory functions.^37–39,58^ The nucleocytoplasmic shuttling properties of AUF1 are mainly related to its nuclear import and export signals,^39^ as well as its interaction with the nuclear export protein CRM1/XPO1.^59^ Further investigation is required to determine how CagA influences these signals of AUF1.

Despite the fact that our experiments are conducted alternately in GES-1 and AGS cell lines as well as human primary gastric epithelial cells, we have noted a high degree of reproducibility across these different cell types. Future studies should endeavor to establish organoid experiments or rodent infection models to advance our understanding. In addition, due to the shortage of antibodies capable of effectively detecting the phosphorylated EPIYA motif within CagA, the phosphorylation status of CagA in human samples cannot be evaluated effectively.

**Figure 8.**
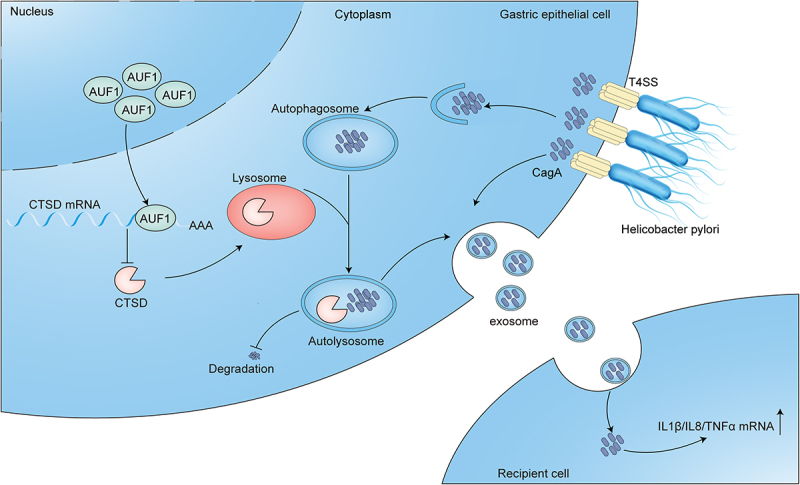
Schematic diagram of AUF1 role during *H. pylori* infection. *H. pylori* CagA upregulates the expression of AUF1 and promotes its distribution to the cytoplasm. At the same time, AUF1 inhibits the autophagic degradation of CagA by inhibiting the expression of CTSD. As a result, the intracellular accumulation of CagA can be secreted, thereby promoting extracellular inflammation.

In summary, this study identified that *H. pylori* CagA could increase the expression of AUF1 and facilitate its translocation to the cytoplasm. Simultaneously, AUF1 inhibited the autophagic degradation of CagA by suppressing CTSD expression. Consequently, the accumulation of intracellular CagA promoted intracellular inflammation and also be secreted in exosomes to induce extracellular inflammation (Figure 8). Our findings provide a novel strategy to attenuate the pathogenic effects of CagA by manipulating autophagic lysosomal degradation pathways of host cells in future studies.

## Materials and methods

### Clinical samples

The human gastric mucosal tissues were collected from the Department of Digestive Pathology of the Peking University Third Hospital (Beijing, China), which contains 15 samples from healthy individuals, 15 samples from patients with *H. pylori*-positive superficial gastritis and 15 samples from patients with *H. pylori*-negative superficial gastritis. Meanwhile, the paired gastric juice samples were collected from the above patients with *H. pylori*-positive or *H. pylori*-negative superficial gastritis. The *H. pylori*‐positive tissues were confirmed by Warthin‐Starry staining.^60^ Moreover, we also collected 45 gastric tissues of individuals of *H. pylori*- infected chronic superficial gastritis with different degrees of inflammation, including mild, moderate and severe (*n* = 15, per group). Diagnoses of all the samples were confirmed histologically by two independent pathologists, and all tissues were assessed by hematoxylin eosin staining. This study was approved by the Medical Ethics Committee of the Peking University Third Hospital (decision numbers M2022684), and written informed consents were signed by all patients.

### H. pylori *culture*

*H. pylori* strain 26695 and CagA knockout *H. pylori* 26695 used in the experiments was from the Key laboratory for *Helicobacter pylori* infection and upper gastrointestinal diseases at Peking University Third Hospital. According to the standard protocol,^23^ *H. pylori* was cultured on Karmali solid medium containing 10% sterile defibrinated sheep blood. *H. pylori* was cultured under microaerobic conditions (5% O_2_, 10% CO_2_, 85% N_2_, 37°C), and the strains were passaged every 2 days.

### Cell culture and infection

Human gastric cancer cell line AGS was maintained from ATCC (Manassas, Virginia, USA). Human gastric epithelial cell line GES-1 was from Shanghai Institute of Cell Biology, Chinese Academy of Sciences (Shanghai, China). Human primary gastric epithelial cells were purchased from Cellverse（iCell）Bioscience Technology from Shanghai, China. AGS and GES-1 cells were cultured in Roswell Park Memorial Institute 1640 medium (HyClone, Logan, Utah, USA) containing 10% fetal bovine serum (Gibco, Waltham, Massachusetts, USA), and 100 U/mL penicillin–streptomycin (Gibco). Human primary gastric epithelial cells were cultured in Primary epithelial cell base medium (Cellverse（iCell）Bioscience Technology, Shanghai, China), containing 2% fetal bovine serum (Cellverse（iCell）Bioscience Technology, Shanghai, China), Primary epithelial cell culture additive (Cellverse（iCell）Bioscience Technology, Shanghai, China), and 100 U/mL penicillin – streptomycin (Cellverse（iCell）Bioscience Technology, Shanghai, China). RAW 264.7 and HEK 293 T cells were cultured in DMEM containing 10% fetal bovine serum and 100 U/mL penicillin–streptomycin. Cells were cultured at 37°C with 5% CO_2_.

The *H. pylori*-cell co-culture experiment was carried out as described previously.^61,62^ Briefly, *H. pylori* collected from the solid medium was washed and resuspended in phosphate buffered saline (PBS). The quantity of *H. pylori* was measured at 600 nm absorbance using a spectrophotometer. Then, cells were infected with *H. pylori* for 24 h at a multiplicity of infection (MOI) of 100:1. The images of *H. pylori* infection and un-infection taken under a bright-field microscope show that each cell could be attached to a certain amount of *H. pylori* (Figure S19).

### Plasmids and cell transfection

Plasmids overexpressing four different isoforms of AUF1 (pCDH-p37 AUF1-3Flag, pCDH-p40 AUF1-3Flag, pCDH-p42 AUF1-3Flag, pCDH-p45 AUF1-3Flag) were kind gifts from Prof. Xiangmei Chen. The overexpression plasmid of CagA (pCDH-CagA-3Flag) was constructed by inserting CagA cDNA into the pCDH-CMV-MCS plasmid (Addgene, Cambridge, MA). Cells were transfected with plasmids using Lipofectamine 2000 transfection reagent (Invitrogen, Shanghai, China) and Opti-MEM (Gibco). The transfection of siRNA targeting AUF1 (siAUF1), CTSD (siCTSD)or siRNA control (siCtrl) was performed using Lipofectamine RNAiMAX Transfection Reagent (Invitrogen, California, USA) by following the manufacturer’s instructions. The sequences of siAUF1 and siCTSD were GTTGTAGACTGCACTCTGA and GCACAGACTCCAAGTATTA，respectively.

### Western blot

Cells were lysed in ice-cold RIPA buffer (Solarbio, Beijing, China) containing protease and phosphatase inhibitors (Solarbio). The protein concentrations were determined by the BCA Protein Assay Reagent (Pierce, Illinois, USA). Protein lysates were separated on NuPAGE Bis-Tris gels (Invitrogen, California, USA) and transferred to a PVDF membrane (Millipore, Massachusetts, USA). The membranes were blocked and probed with antibodies. Detailed information regarding the antibodies used in this study is provided in Table S2. Enhanced chemiluminescence reagent (Yamei, Shanghai, China) or the Odyssey detection system (LI-COR, Lincoln, Nebraska, USA) were used to detect protein expression. Gray values were analyzed by ImageJ 1.52a software.

### Quantitative real-time PCR (qRT-PCR) analysis

Total RNA was isolated using Trizol (Invitrogen, California, USA) and reversely transcripted to cDNA by Super Script First-Strand Synthesis System (Roche, Basel, Switzerl). qRT-PCR analysis was performed in Roche 480 using SYBR Green I Master (Roche, Basel, Switzerland). Primer sequences used in this study are listed in Table S3. *β-Actin* gene was used as an internal control and the relative expression of target genes was calculated using the 2–ΔΔCt method.

### RNA turnover assay

RNA turnover assay was used to measure the half-life of mRNA. AGS cells were transfected with control or AUF1 siRNA for 48 h followed by adding Actinomycin D to inhibit transcription (5 mg/mL, Abcam, Cambridge, UK) into the cell culture medium. At 0, 1, 2, 3, 4, 5 h post treatment, RNA was prepared using Cell-to-CT™ Kit (Invitrogen, California, USA) and subjected to qRT-PCR analysis using specific primers.

### RNA immunoprecipitation (RIP) assay

RNA immunoprecipitation was performed as previously described.^63^ Briefly, a total of 1 × 10^7^ AGS cells were lysed with RIP lysis buffer. The cell lysates were incubated with AUF1 antibody or isotype-matched control antibody embedded beads overnight at 4°C. Then the beads were washed and resuspended using NT2 buffer with RNase-free DNase I (Roche, Basel, Switzerl) and proteinase K (Invitrogen, California, USA). The coprecipitated RNAs in the NT2 buffer were isolated by using phenol chloroform extraction and ethanol precipitation. The target transcripts were measured by qRT-PCR.

### RNA pulldown assay

For biotin-based RNA pulldown assays, PCR-amplified DNA was used as the template to transcribe biotinylated RNA by using T7 RNA polymerase in the presence of biotin-UTP (Biotium, CA, USA), as described previously.^64^ And 1 μg of purified biotinylated transcripts was incubated with 100 μg total cell lysates for 30 min at room temperature and then mixed with streptavidin-Dyna beads (Invitrogen, California, USA). After the beads were thoroughly washed, the pulldown material was analyzed by western blot assay.

### Immunohistochemistry analysis

Anti-AUF1 and anti-CTSD were used for detection as previously described,^23^ following the instructions of the antibodies. In brief, tissue samples were rehydrated and antigen was collected in citrate buffer (Anti-AUF1, pH 6.0) or in Tris-EDTA buffer (anti-CTSD, pH 9.0) with microwave treatment after deparaffinization. Then, incubate sections in 3% hydrogen peroxide for 10 min and blocking was performed with antibody diluent (ZLI-9056, ZSGB-BIO, China), followed by incubation with primary antibody for 2 h at 37°C, detection using HRP Ms + Rb secondary antibody, and visualization using DAB chromogens. Six fields were randomly selected for analysis by ImageJ 1.52a software (Silver Spring, Maryland, USA). Average number of the positive area was compared by paired *t*-tests.

### Exterogenous overexpression of CagA degradation assay

After transfected with pCDH-CagA-3Flag plasmids for 48 h, GES-1 cells were treated with 25 μg/mL cycloheximide to inhibit protein synthesis (CHX, Selleck, Shanghai, China). At the 0, 1, 3, 6, 9 h, cells were washed with PBS and collected. The remaining CagA was measured by western blotting as described above.

### H. pylori *CagA degradation assay*

AGS cells were washed three times in PBS after being infected with *H. pylori* for 5 h. Then the cells were incubated at 1 h in a medium containing gentamicin (100 μg/mL) to kill extracellular bacteria. At the 0, 3, 6, 9, 15, 24 h, cells were washed with PBS and collected. The remaining CagA and p-Tyr was measured by western blotting as described above.

### Immunofluorescence

GES-1 cells were seeded on a glass culture dish. The cells were fixed with paraformaldehyde for 15 min. After the cells were permeabilized by 1% TritonX-100 for 15 min at RT, cells were blocked with 5% bovine serum albumin for 1 h. Cells were then incubated with the primary antibodies at 4°C for 16 h, washed, and incubated with the secondary antibodies for 1 h at RT. Cells were stained with Hoechst 33342 (Beyotime Biotechnology, China) for 10 min at RT. Images were acquired by confocal microscope (Leica TCS SP8 X).

To detect GFP-LC3B/mCherry-GFP-LC3B signals, AGS cells treated with control or AUF1 siRNA or AUF1 overexpressing Plasmids for 6 h were infected by the AD-GFP-LC3B (Beyotime Biotechnology, China) or AD-mCherry-GFP-LC3B (Beyotime Biotechnology, China) for 24 h at MOI of 50:1. And then AGS cells were infected with *H. pylori* for 6 h. The cells were incubated with RPMI1640 culture medium containing 400 μg/ml kanamycin for 24 h, then fixed with 4% paraformaldehyde. After being permeabilized and blocked, images were acquired by confocal microscope (Leica TCS SP8 X).

To detect the LysoTracker Red/LysoSensor Yellow/Blue DND-160 signals, after *H. pylori* infection for 6 h, the AGS cells with control or AUF1 siRNA for 24 h were incubated with RPMI1640 culture medium containing 400 μg/ml kanamycin for 24 h, and then with LysoTracker Red DND-99 (Invitrogen)/LysoSensor Yellow/Blue DND-160 (MedChemExpress, HY-D1445) for 90 min. After washing off the dye, images were acquired by confocal microscope (Leica TCS SP8 X).

### Transcriptomic sequencing

AGS cells were transfected with control or AUF1 siRNA for 24 h. Then, cells were infected with *H. pylori* for 24 h at a multiplicity of infection of 100:1. Wash the cells thrice with cold PBS and collect the pellets by centrifuging at 800 rpm for 4 min, and then send the pellets to Novogene Biology (Beijing, China) for transcriptomic sequencing. Hisat2 and featureCounts were used for reads mapping and gene count calculating. The mapped read counts were counts per million (CPM) normalized, and differential gene expression profile analysis was performed with Edge R with the screening threshold set as follows: *p* < 0.05 and |log2(Fold Change) |>0.5. GO function analysis provides classification annotation and a significant enrichment analysis of the differential genes. Cluster analysis and hypergeometric test were used as statistical tests to carry out KEGG pathway enrichment analysis of the selected differential genes.

### Calcium assays

Fura-4 AM (Beyotime Biotechnology, China) was applied to determine cytosolic Ca^2+^ signals. Cells grown in a 96-well plate and the number of cells per well needs to be controlled in the range of 2000–5000. After being washed three times with PBS, cells were loaded with 2 µM Fura-4AM for 30 min at room temperature. After incubation, cells also need be washed 3 times with PBS. Then, after being treated with Gly-Phe-*β*-naphthylamide (GPN, hydrolyzed by CTSC leading to a loss of lysosome membrane integrity) or DMSO,^26^ fluorescence signal of Fura-4 AM was detected by Multimode Plate Reader (PerkinElmer, Ex/Em = 490/525 nm).

### Exosomes isolation and identification

AGS cells were cultured in normal medium until they were 80% confluent. Then the medium was replaced with exosome-depleted medium (RPMI1640 containing 10% exosome-depleted FBS). Two days later, the cell supernatant was collected and ultracentrifugation to harvest exosomes. As described previously,^65,66^ the conditioned medium was successively centrifuged at 3,00 × *g* for 10 min, 2,000 × *g* for 10 min, and 10,000 × *g* for 30 min to remove cell fragments, cell debris, and large extracellular vesicles. Finally, exosomes were then harvested at 110,000 × *g* for 120 min. The pellet was resuspended in particle-free PBS or stored at −80°C in the refrigerator for subsequent use. For exosome purification from gastric juice, first, gastric juice was filtered through a 0.23 mm-mesh for removing residues and then was filtered through a 0.22-μm pore filter according to the standard protocol.^53,67^ Finally, exosomes were then harvested at 110,000 × *g* for 120 min.

### Particle size distribution and quantification

Size distribution and concentration of exosomes were determined by NTA using a NanoSight LM10 HS instrument equipped with a NanoSight LM14 unit with on-board temperature control (Malvern Panalytical Ltd., Malvern, UK), LM 14C (405 nm, 65 mW) laser unit and high sensitivity camera with a Scientific CMOS sensor (C11440-50B, Hamamatsu Photonics, Hamamatsu City, Japan). Six 60-s videos were recorded for two independent replicates, generating 12 individual measurements for each sample. Further processing was performed as we described previously.^66^

### Transmission electron microscope (TEM) analysis

For exosome observation, exosome was resuspended in 4% paraformaldehyde (PFA), EV’s morphology analysis was performed using a JEM-1011 transmission electron microscope (JEM-1400PLUS, Japan) at 60 kV according to the protocol described in Skryabin et al.^66^ For autophagosome and autophagolysosome, cells were fixed in 2.5% glutaraldehyde at 4°C overnight and postfixed with 2% osmium tetroxide for 1.5 h at room temperature. After fixation, cells were embedded and stained. Morphological observation was examined under a transmission electron microscope (JEM-1400PLUS, Japan) at 60 kV. Photos of at least five fields of view were collected and analyzed for each sample.

### Exosome labeling and tracking

In vitro experiment, a total of 10 μg purified exosomes were labeled with a PKH26 kit (Sigma-Aldrich, USA) according to the manufacturer’s instructions. GES-1 or RAW264.7 cells were plated on confocal dishes and incubated with PKH26-labeled exosomes or PKH26 dye as a negative control for 0, 6, 12, 24 h at 37°C. The cells were washed and fixed, visualized with a confocal microscope (Leica TCS SP8 X).

### Assessment of CagA, IL-1β and TNF-α levels in gastric juice and cell supernatant

According to the manufacturer’s instructions, the concentrations of CagA levels in cell supernatant and gastric juice were determined using enzyme-linked immunosorbent assay (ELISA; MEIMIAN, MM-1173H2, China). The exosomes of cell supernatants and gastric juice were purified as described above, and then CagA levels were assessed according to the manufacturer’s instructions. The concentrations of IL-1β and TNF-α levels in gastric juice were determined using enzyme-linked immunosorbent assay (ELISA; MLBIO, ML058059V-96T and ML064303-C-96T, China).

### Statistical analysis

Statistical analyses were performed using SPSS software 27.0 (Chicago, Illinois, USA). Two-tailed Student’s *t*-test or one-way ANOVA was used to evaluate significant differences. *p* < 0.05 was considered statistically significant.

## Supplementary Material

Supplemental Material

## Data Availability

The datasets generated and analyzed during the current study are available from the corresponding author on reasonable request.
