# Supplementary material for: AUF1-mediated inhibition of autophagic lysosomal degradation contributes to CagA stability and Helicobacter pylori-induced inflammation
Source: Gut Microbes. 2024 Jul 28;16(1):2382766. doi: 10.1080/19490976.2024.2382766 (PMC11285221; doi:10.1080/19490976.2024.2382766)
Supplement: Supplemental Material [file KGMI_A_2382766_SM7003.zip › Revised_supplementary_Figures.docx]

# Supplementary Figures

**Fig S1**


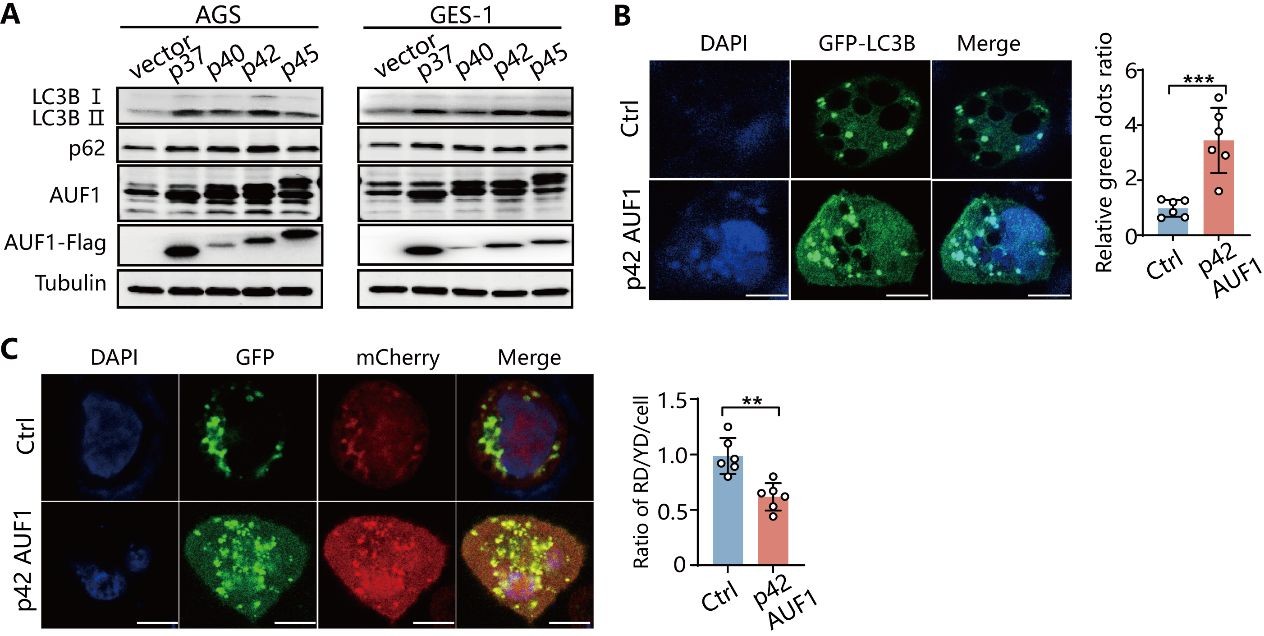


Fig S1. AUF1 inhibited autophagic flux. (A). LC3B and p62 expression was analyzed by Western blot assays in AGS and GES-1 cells with AUF1 overexpression. (B). The representative images illustrate GFP-LC3 reporter signals by IF assays in AGS cells. AGS cells treated with AUF1 overexpression were infected by the AD-GFP-LC3 at MOI of 50:1. And then AGS cells were infected with *H. pylori* for 24h. Scale bar 5 μm. The experiment was independently repeated three times. Immunofluorescence intensity of green dots in cell was analyzed by ImageJ. Data are presented as the mean ± SD of 6 independent images. ****p* < 0.001. (C). The representative images illustrate mCherry-GFP-LC3 reporter signals by IF assays in AGS cells. AGS cells transfected with empty vector or p42-AUF1 overexpressing plasmids were infected by the AD-mCherry-GFP-LC3 at MOI of 50:1. The experiment was independently repeated three times. And then AGS cells were infected with *H. pylori* for 24h. Scale bar 5 μm. Immunofluorescence intensity of red dots (RD) and green dots (GD) in cell was analyzed by ImageJ. Data are presented as the mean ± SD of 6 independent images. ***p* < 0.01.

# Fig S2


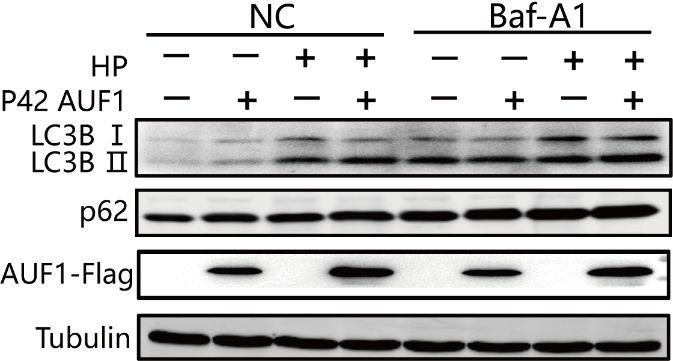


Fig S2. AUF1 inhibited autolysosomal degradation. After treatment with 10μM Baf A1, LC3B and p62 expression was analyzed by Western blot assays in AGS cells transfected with empty vector or p42-AUF1 overexpressing plasmids during un-infection or *H. pylori* infection.

# Fig S3


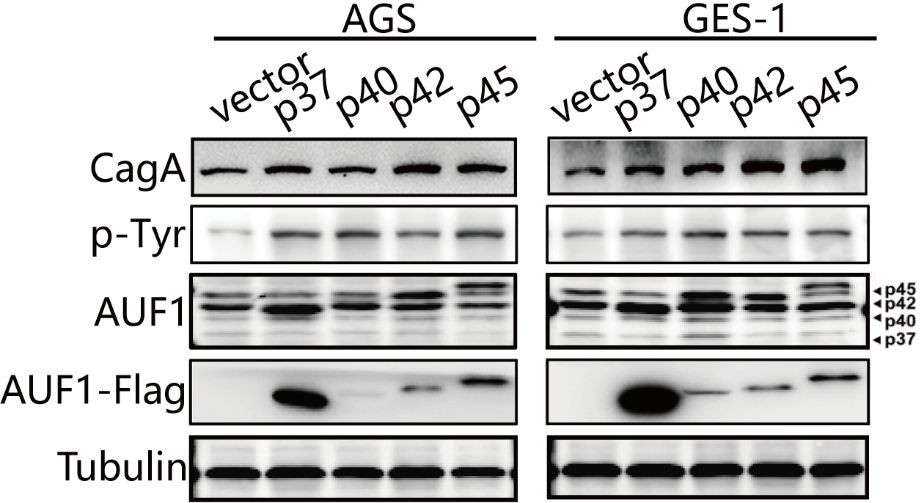


Fig S3. AUF1 caused intracellular cagA accumulation. CagA and p-Tyr expression was analyzed by Western blot assays in *H. pylori* -infected AGS cells and GES-1 cells after AUF1 overexpression or not.

#
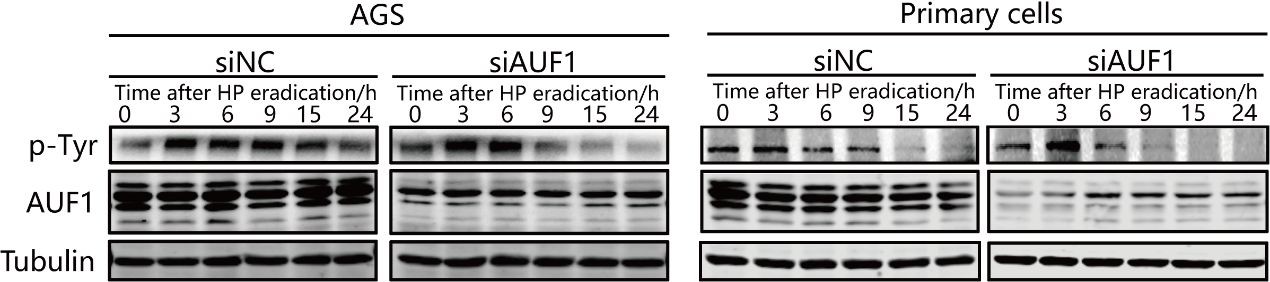
Fig S4

Fig S4. After 5 h of *H. pylori* 26695 infection, the AGS cells and primary gastric epithelial cells with AUF1 knockdown were incubated with gentamicin for 1h to kill extracellular bacteria. Subsequently, p-Tyr expression at indicated times was analyzed by Western blot assays.

# Fig S5


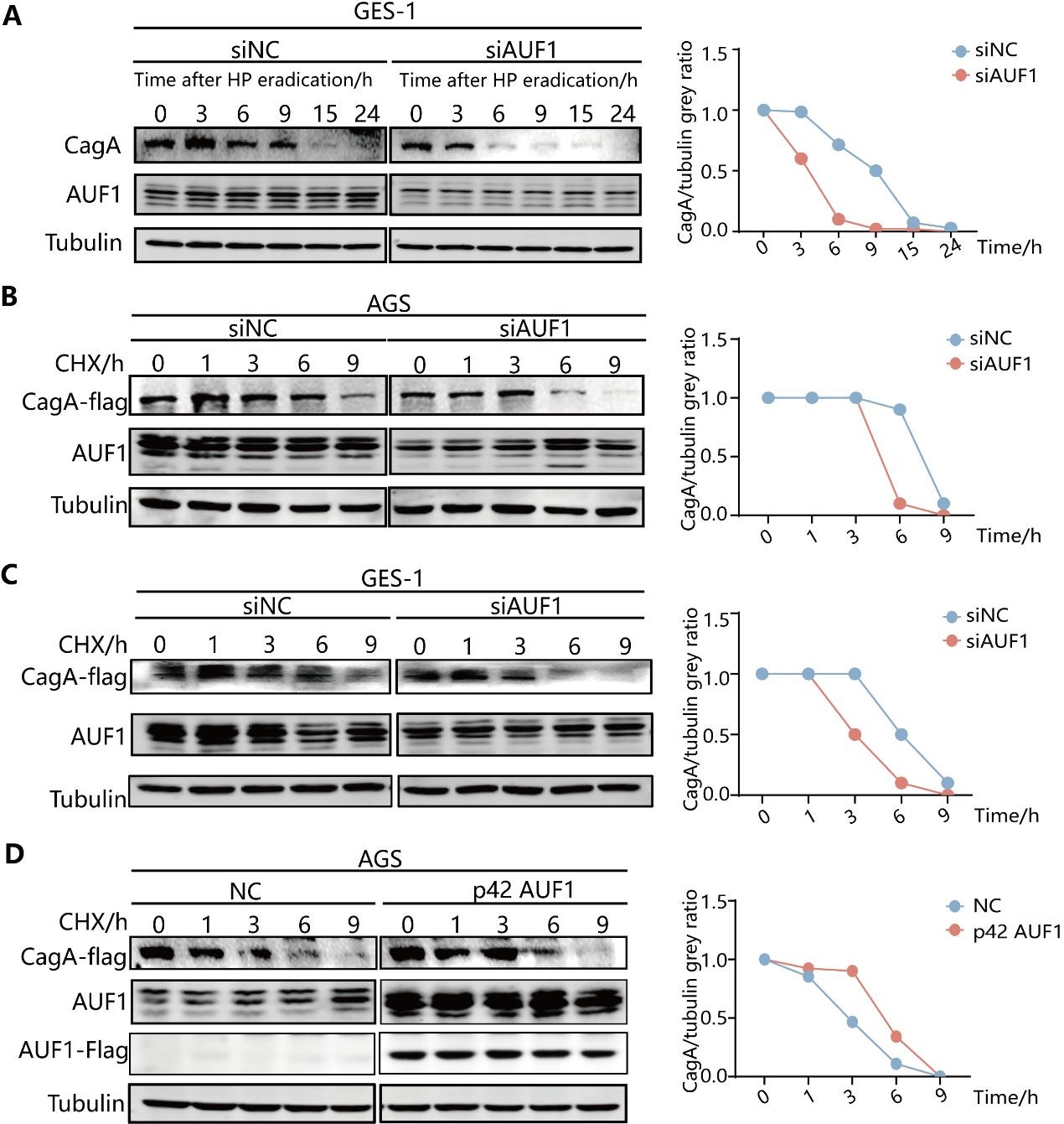


Fig S5. AUF1 inhibited CagA degradation. (A). After 5 h of *H. pylori* 26695 infection, the GES- 1 cells with AUF1 knockdown or not were incubated with gentamicin for 1h to kill extracellular bacteria. Subsequently, CagA expression at indicated times was analyzed by Western blot assays, gray values of CagA band were analyzed by ImageJ and CagA/tubulin gray ratio was quantified. CagA/tubulin gray ratio at indicated times was quantified. (B&C). After transfected with exogenous CagA-overexpressing clone plasmid for 24h in AGS cells(B) or GES-1 cells(C) with AUF1 knockdown following incubation with cycloheximide (CHX, 25 µg/mL), CagA expression at indicated times was analyzed by Western blot assays. Gray values of CagA band were analyzed by ImageJ and CagA/tubulin gray ratio was quantified. (D). After transfected with exogenous CagA- overexpressing clone plasmid in AGS cells with p42-AUF1 overexpression, CagA expression at indicated times was analyzed by Western blot assays. Gray values of CagA band were analyzed by ImageJ and CagA/tubulin gray ratio was quantified.

#
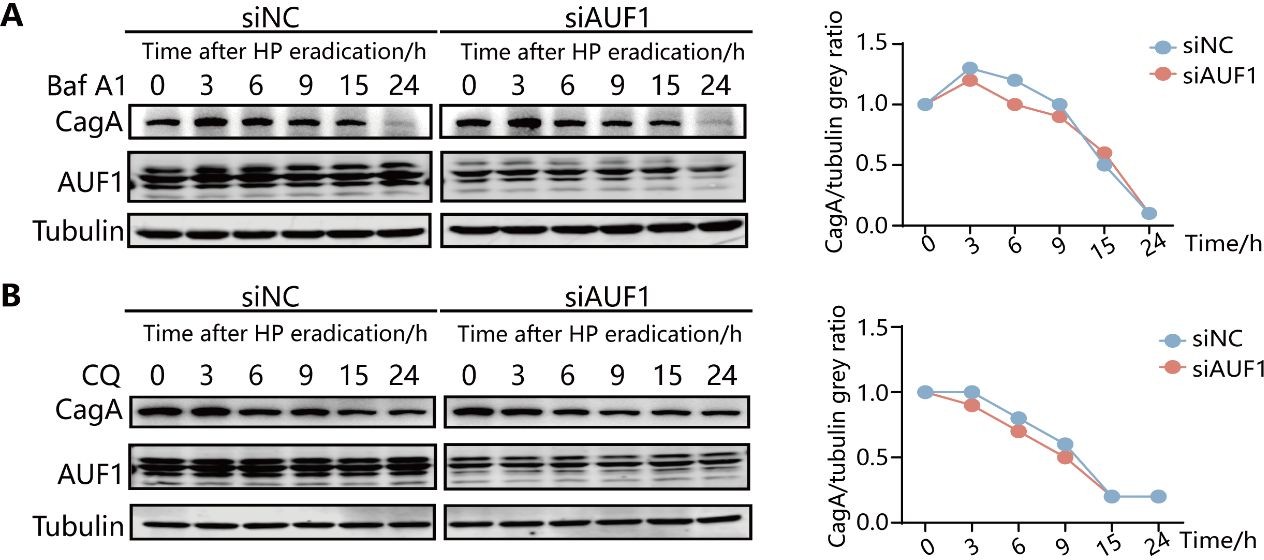
Fig S6

Fig S6. BafA1 and CQ can abolish the effect of AUF1 knockdown in promoting CagA degradation. (A&B). After treatment with 10μM Baf A1(A) or 50μM CQ(B), CagA expression at indicated times was analyzed by Western blot assays in AGS cells with AUF1 knockdown after the eradication of *H. pylori*, gray values of CagA band were analyzed by ImageJ and CagA/tubulin gray ratio was quantified.

# Fig S7


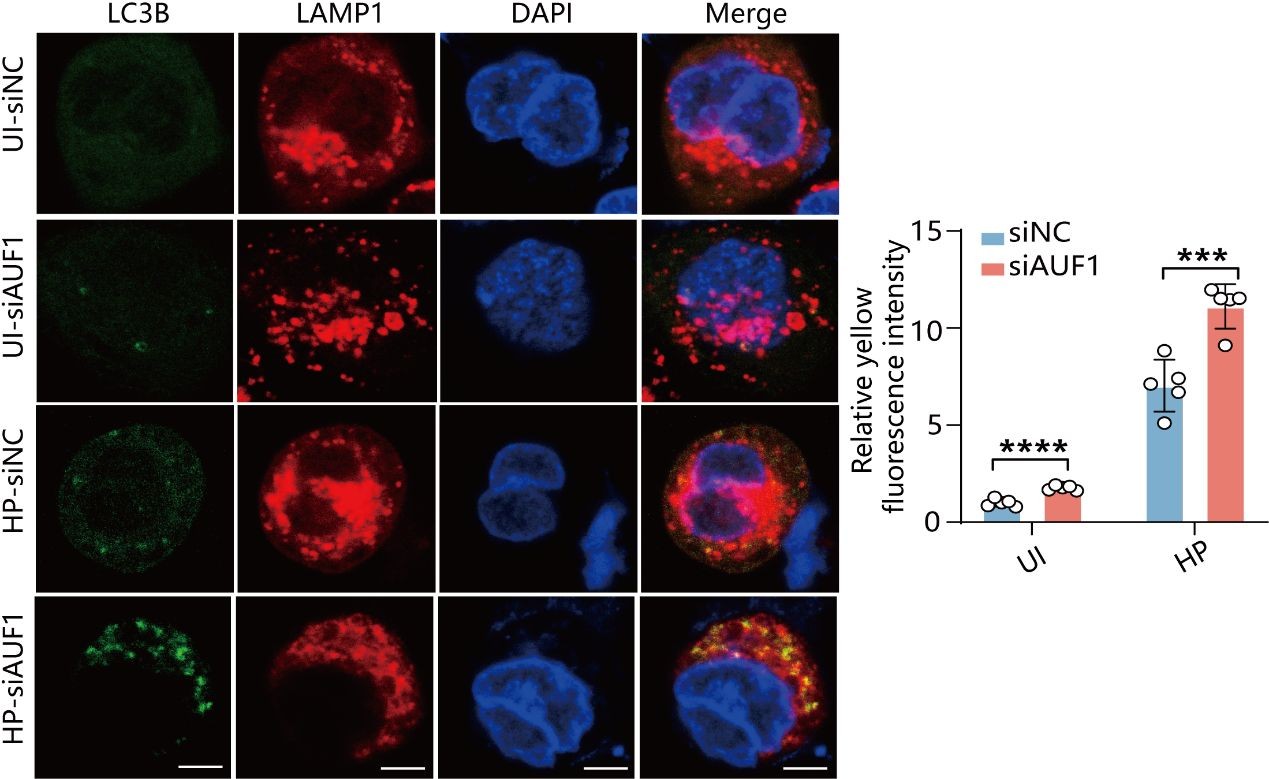


Fig S7. AUF1 didn’t affect autophagosome-lysosomal fusion. GFP-LC3 and LAMP1 was analyzed in AGS cells with AUF1 knockdown during *H. pylori* infection by immunofluorescence assay and yellow fluorescence intensity was quantified by ImageJ. The experiment was independently repeated three times. Scale bar 5 μm. Data are presented as the mean ± SD of 5 independent images.

****p* < 0.001, *****p* < 0.0001.

# Fig S8


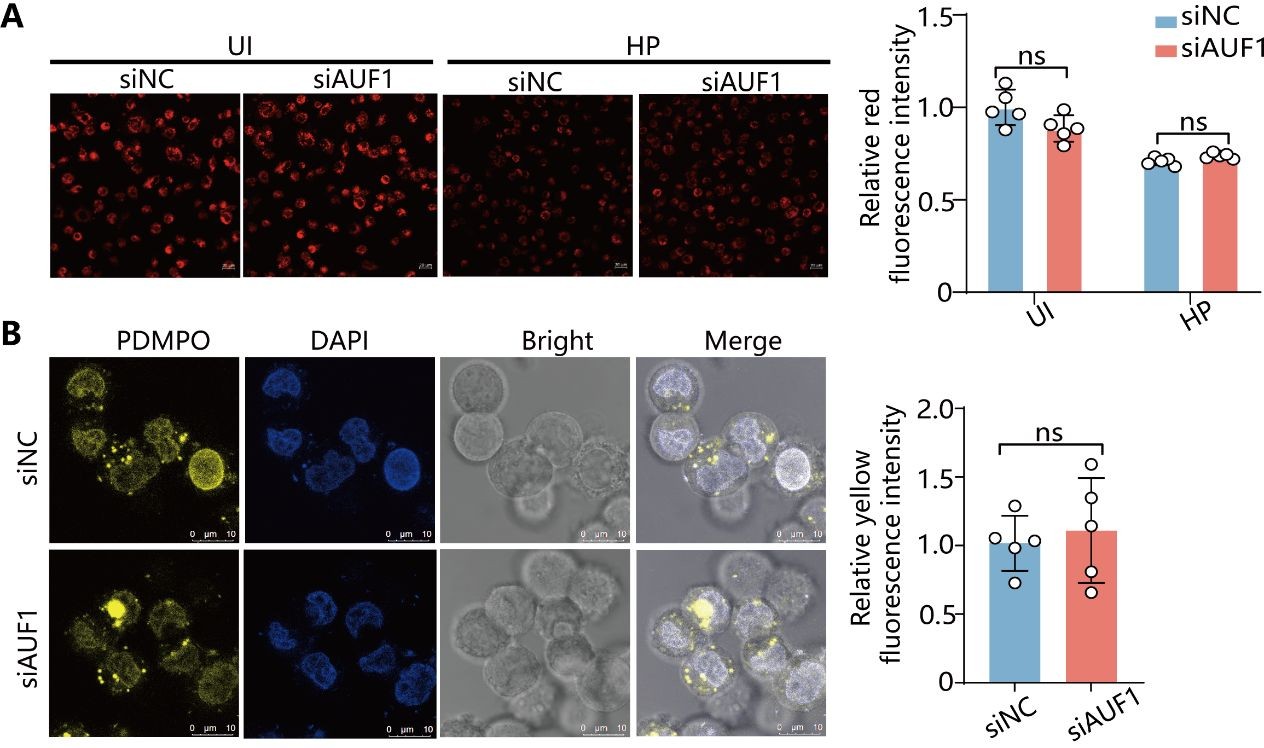


Fig S8. AUF1 didn’t affect lysosomal acidity. AGS cells with AUF1 knockdown during un-infection or *H. pylori* infection were stained with LysoTracker Red(A) / LysoSensor Yellow/Blue DND- 160(B) to assess lysosomal activities. The experiment was independently repeated three times. Immuno-fluorescence intensity in cell was analyzed by ImageJ. Data are presented as the mean ± SD of 5 independent images, ns means not significant.

# Fig S9


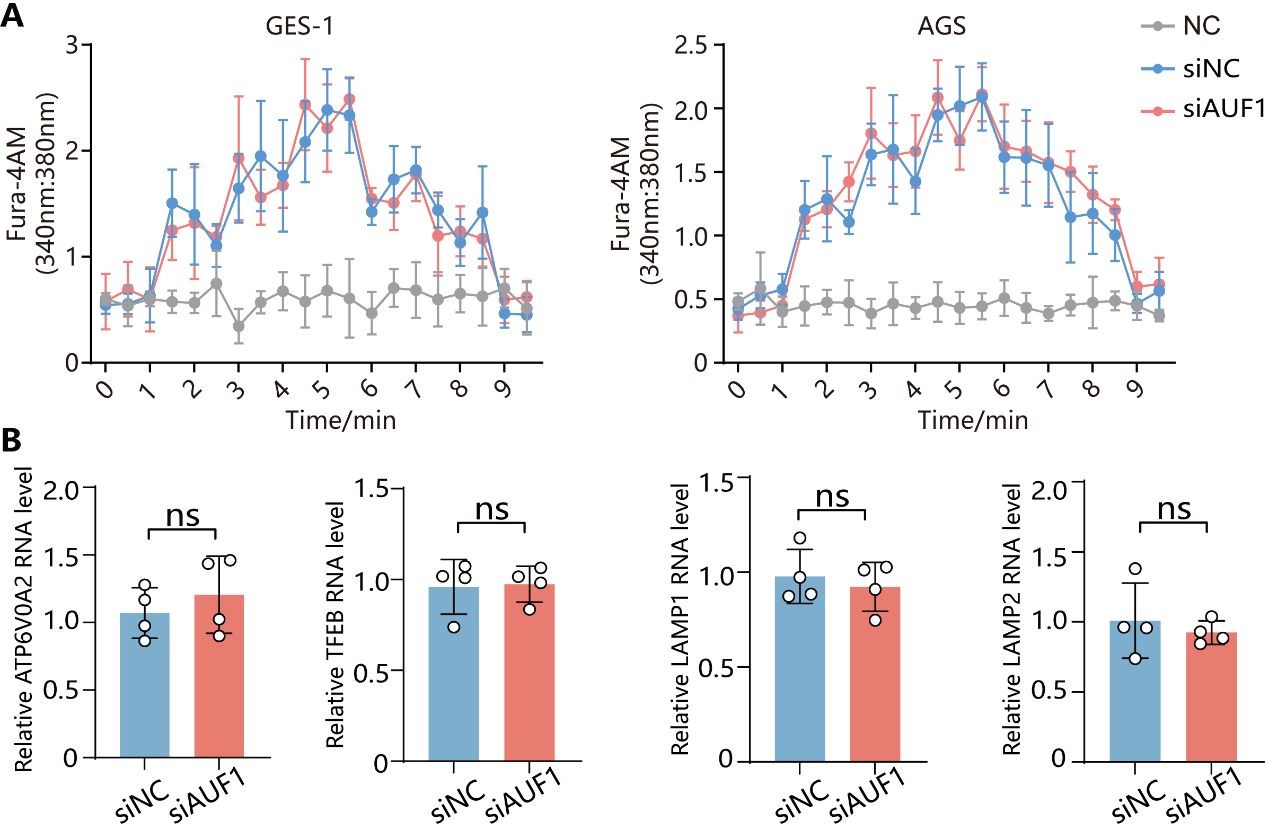


Fig S9. AUF1 didn’t affect Ca2+ release and the expression of some key lysosomal degradation- related genes. (A). Ca2+ release at indicated times was analyzed by Fluo-4AM assays in GES-1 and AGS cells with AUF1 knockdown. (B). ATPV0A2, TFEB, LAMP1/2 mRNA was analyzed by RT- qPCR in AGS cells with AUF1 knockdown during un-infection or *H. pylori* infection. Data is expressed as means ± SD from 3 independent experiments, ns means not significant.

# Fig S10


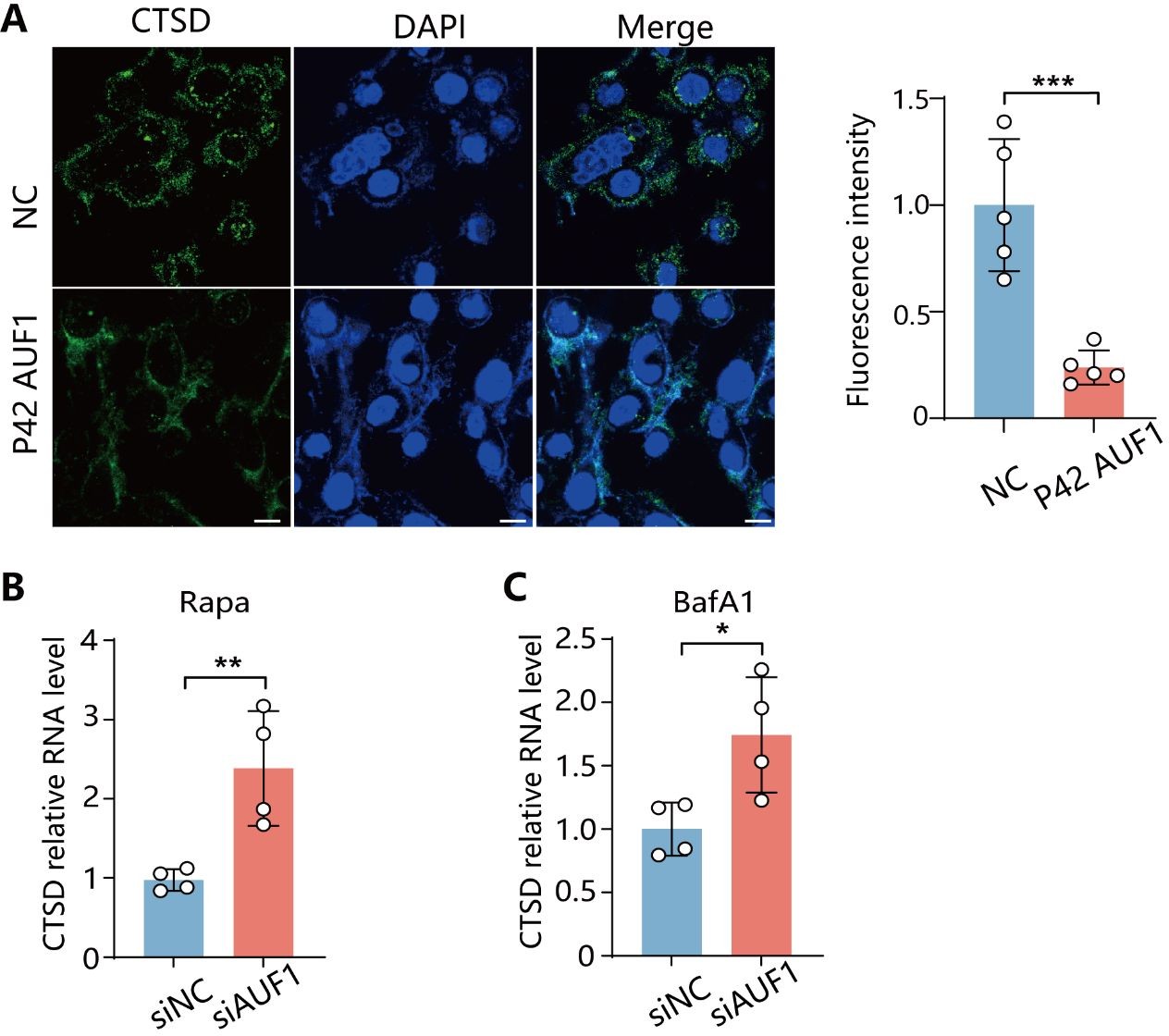


Fig S10. AUF1 inhibited the expression of CTSD. (A). CTSD levels were analyzed by IF assay in

*H. pylori*-infected AGS cells with AUF1 overexpression. Scale bar 10 μm. The experiment was independently repeated three times. Immunofluorescence intensity of red and green in cell was analyzed by ImageJ. Data are presented as the mean ± SD of 5 independent images. ****p* < 0.001. (B&C). After treatment with Rapa(B) or BafA1(C), CTSD mRNA was analyzed by RT-qPCR in AGS cells with AUF1 knockdown during *H. pylori* infection. Data is expressed as means ± SD from 3 independent experiments. **p* < 0.05, ***p* < 0.01.

# Fig S11


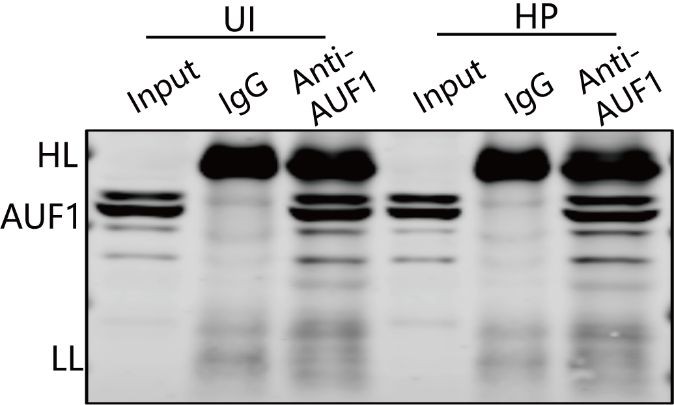


Fig S11. IP efficiency verification of RIP assays. AUF1 was analyzed in AGS cells during un- infection or *H. pylori* infection with immunoprecipitating with anti-AUF1 antibody or anti-IgG antibody by Western blot assays.

# Fig S12


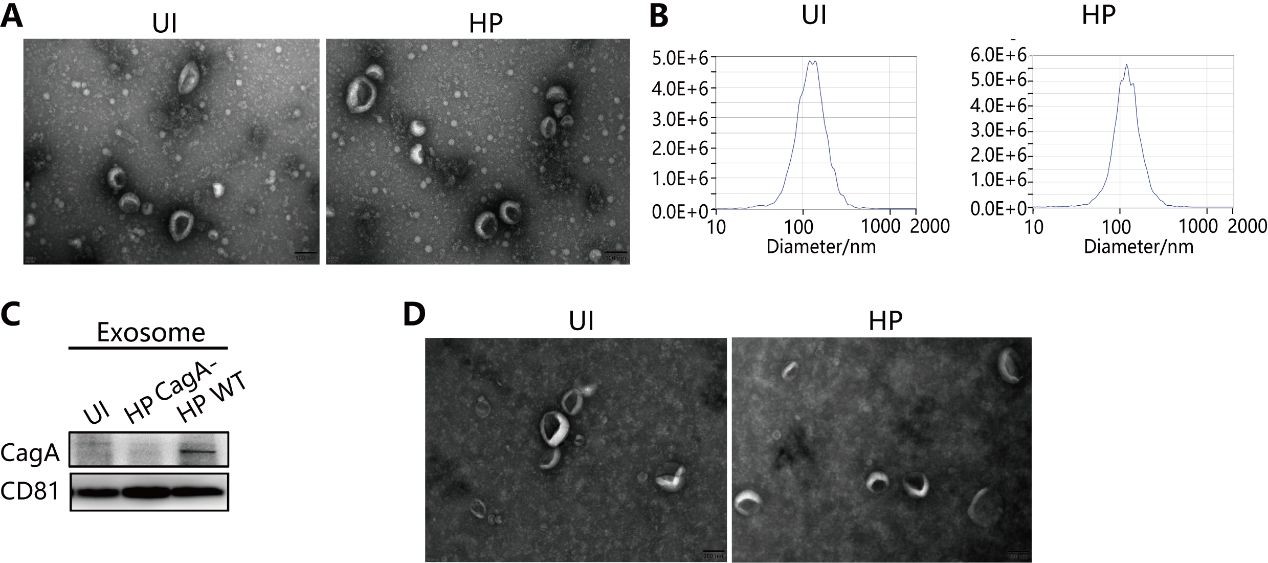


Fig S12. Exosomes isolation and identification in AGS cells. (A). Ultrastructural features of exosomes in AGS cells supernatant during *H. pylori* infection or not were analyzed by transmission electron microscopy. (B). Diameter distribution of exosomes vesicles in AGS cells supernatant during *H. pylori* infection or not were analyzed by NTA. (C). AGS cells were infected with wild- type or CagA-knockout *H. pylori* or uninfected for 48h, then the cell supernatant was collected and purified to harvest exosomes by ultracentrifugation. Western blot assay was performed to detect the expression of CagA in exosomes of cell supernatant. The whole experiment was repeated independently for three times each. (D). Ultrastructural features of gastric juice exosomes in clinical superficial gastritis patients with *H. pylori* infection or un-infection were analyzed by transmission electron microscopy.

# Fig S13


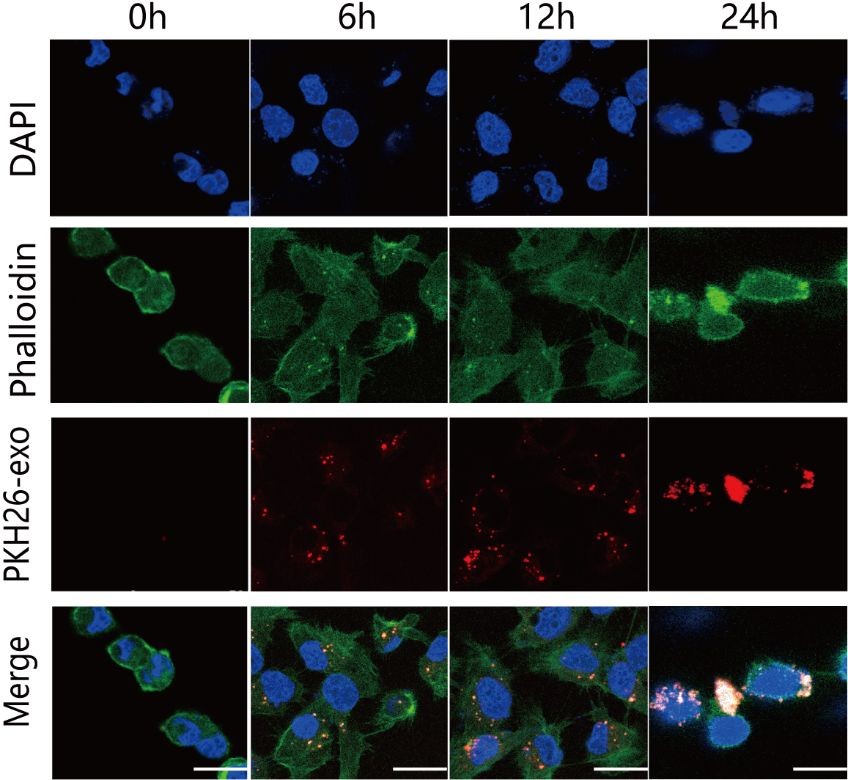


Fig S13. AGS-derived exosomes during *H. pylori* infection were labelled with PKH26 dye to observe its entry into GES-1 cells by IF assay. Scale bar 25 μm.

#
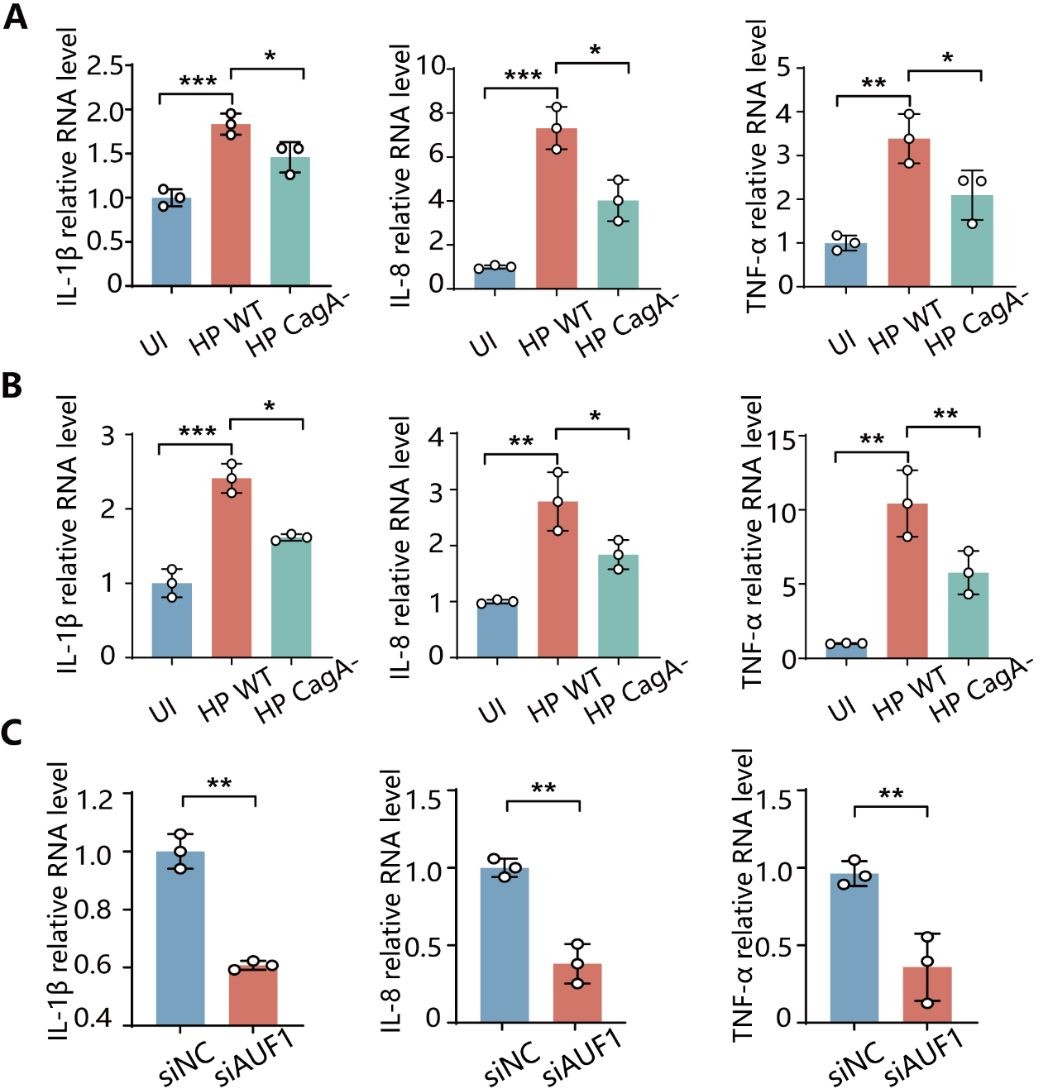
Fig S14

Fig S14. AUF1 can promote secretion of CagA exosomes and up-regulate extracellular inflammatory cytokine expression. IL-1β, IL-8 and TNF-α mRNA level in GES-1 cells (A) and RAW264.7 cells (B) incubating with exosomes from AGS cells with un-infection or wild *H. pylori* or CagA-knockout *H. pylori* infection. (C). IL-1β, IL-8 and TNF-α mRNA level in Raw264.7 cells incubating with exosomes from *H. pylori*-infected AGS cells with AUF1 knockdown. Data is expressed as means ± SD of 3 independent tests. **p* < 0.05, ***p* < 0.01, *****p* < 0.0001.

# Fig S15


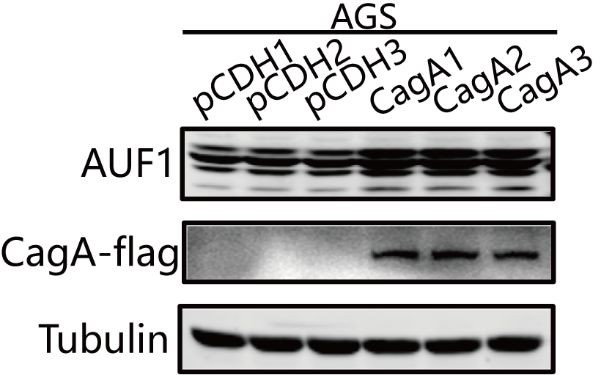


Fig S15. Exogenous CagA upregulated AUF1 protein levels in CagA-overexpressing AGS cells. Constructed different exogenous CagA-overexpressing plasmid was transfected into AGS cells. AUF1 expression was analyzed by Western blot assays.

# Fig S16


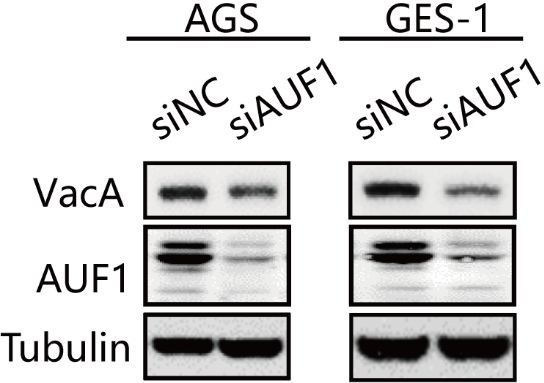


Fig S16. VacA expression was analyzed by Western blot assays in *H. pylori* -infected AGS cells and GES-1 cells after AUF1 knockdown or not.

# Fig S17


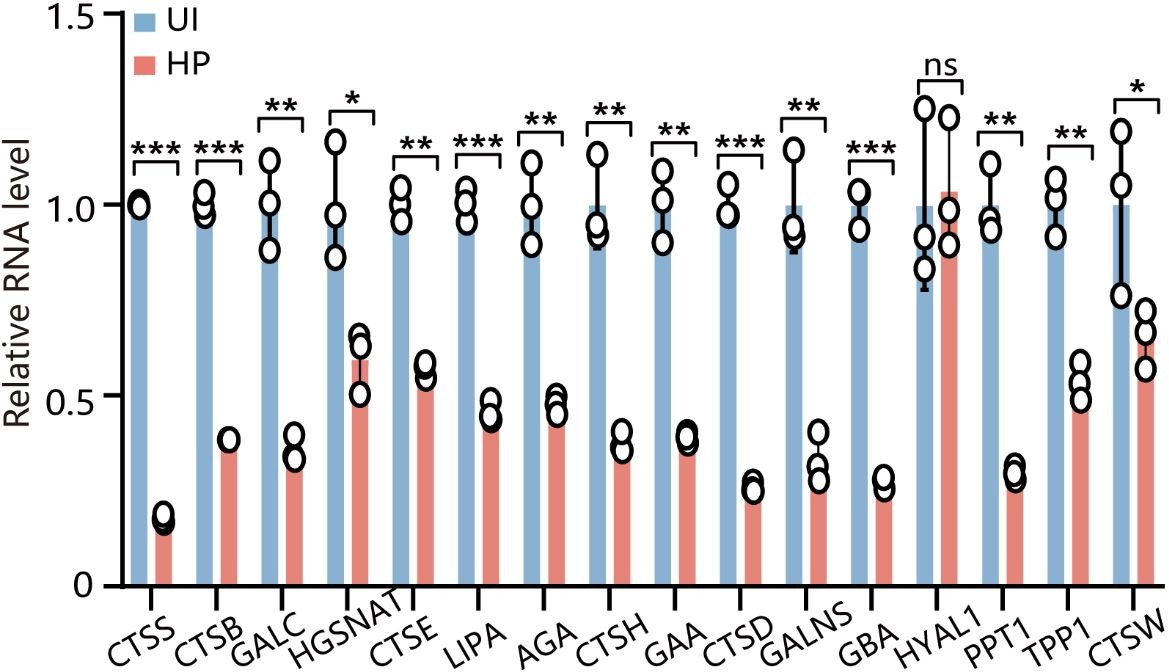


Fig S17. Identified 16 highly enriched lysosomal hydrolases genes mRNA was analyzed by RT- qPCR in AGS cells during *H. pylori* infection. Data is expressed as means ± SD from 3 independent experiments. **p* < 0.01, ***p* < 0.01, ****p* < 0.001, *****p* < 0.0001, ns means not significant.

# Fig S18


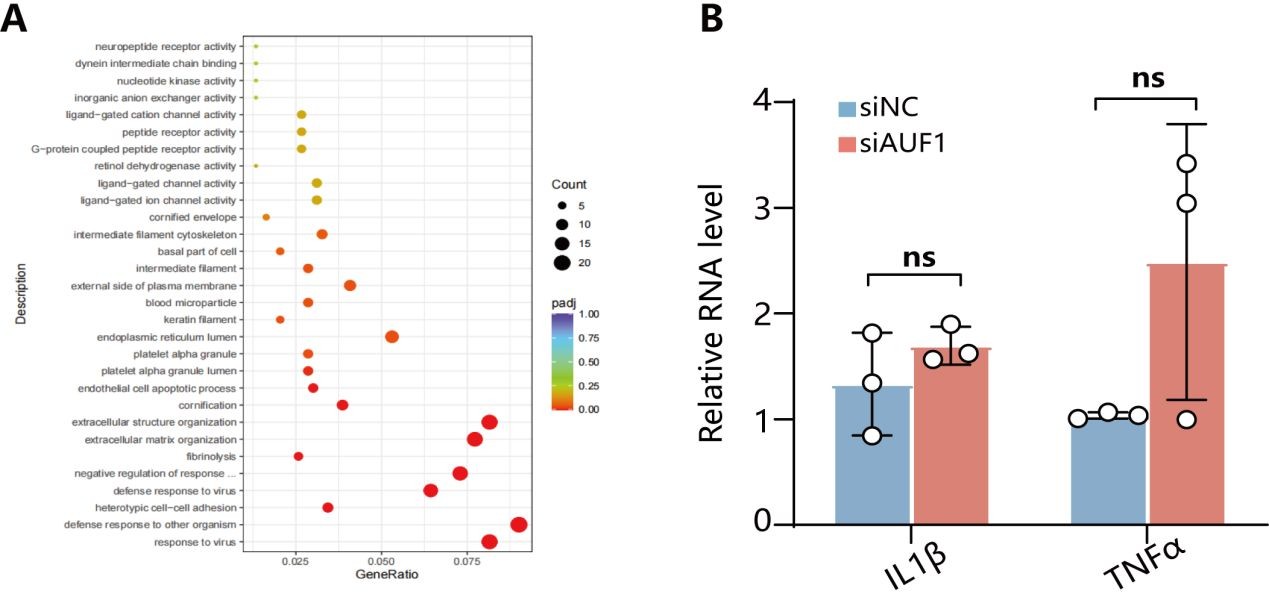


Fig S18. Transcriptome analysis in AGS cells with AUF1 knockdown. (A). GO analysis in transcriptomics data of AGS cells with AUF1 knockdown. (B). IL-1β and TNF-α mRNA level in AGS cells with AUF1 knockdown according to the transcriptome analysis in AGS cells with AUF1

knockdown. Data is expressed as means ± SD of 3 independent tests, ns means not significant.

# Fig S19


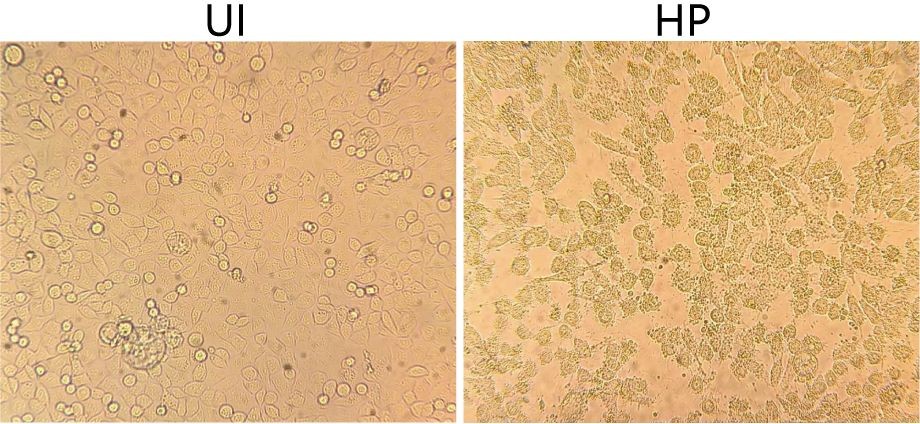


Fig S19. The morphological changes of AGS cells were observed under microscope bright field with un-infection and *H. pylori*-infection for 24h.
