# Supplementary material for: AUF1-mediated inhibition of autophagic lysosomal degradation contributes to CagA stability and Helicobacter pylori-induced inflammation
Source: Gut Microbes. 2024 Jul 28;16(1):2382766. doi: 10.1080/19490976.2024.2382766 (PMC11285221; doi:10.1080/19490976.2024.2382766)
Supplement: Supplemental Material [file KGMI_A_2382766_SM7003.zip › Revised_supplementary_Tables.docx]

Supplementary Tables

**Table S1.** The top ten enrichment pathways of KEGG pathway analysis based on GO analysis of 557 “catalytic activity” related genes in molecular function

| ID | Term | Term *p*  Value | % Associated  Genes | Nr.  Genes | Associated Genes Found |
| --- | --- | --- | --- | --- | --- |
| KEGG:04142 | Lysosome | 0.00011 | 12.12 | 16.00 | AGA, CTSB, CTSD, CTSE, CTSH, CTSS, CTSW,  GAA, GALC, GALNS, GBA, HGSNAT, HYAL1, LIPA, PPT1, TPP1 |
| KEGG:00230 | Purine metabolism | 0.00027 | 11.72 | 15.00 | ADCY9, ADPRM, AK7, ENPP4, ENTPD8, GMPR2,  HDDC2, HPRT1, LACC1, NPR2, NUDT2, PFAS, PGM1, PRPS1, RRM2 |
| KEGG:04071 | Sphingolipid signaling pathway | 0.00041 | 11.76 | 14.00 | CTSD, GNA13, GNAI2, MAPK1, MAPK11, MAPK9, NOS3, PLCB4, PRKCZ, RAC2, RAC3, SGPP1, SGPP2,  SPHK1 |
| KEGG:04210 | Apoptosis | 0.00051 | 11.03 | 15.00 | BIRC3, CASP2, CASP3, CASP7, CTSB, CTSD, CTSH, CTSS, CTSW, ENDOG, GZMB, MAPK1, MAPK9, PARP1, PTPN13 |
| KEGG:04010 | MAPK signaling pathway | 0.00150 | 8.16 | 24.00 | CASP3, DUSP2, DUSP6, DUSP7, HSPA1A, HSPA2, HSPA6, IL1R1, MAP3K12, MAP3K20, MAPK1, MAPK11, MAPK7, MAPK9, MECOM, MET, PDGFRA, PLA2G4C, PPP3CB, PTPRR, RAC2, RAC3,  RPS6KA5, STK4 |
| KEGG:05418 | Fluid shear stress  and atherosclerosis | 0.00190 | 10.07 | 14.00 | [ASS1, GSTM1, GSTM4, HSP90AA1, IL1R1,  MAPK11, MAPK7, MAPK9, MGST1, NOS3, PLAT, PRKCZ, RAC2, RAC3] |
| KEGG:05417 | Lipid and atherosclerosis | 0.01357 | 7.91 | 17.00 | CASP3, CASP7, HSP90AA1, HSPA1A, HSPA2,  HSPA6, IKBKE, JAK2, MAPK1, MAPK11, MAPK9, MMP1, NOS3, PLCB4, PPP3CB, SOD2, TLR6 |
| KEGG:05170 | Human  immunodeficiency virus 1 infection | 0.07985 | 6.60 | 14.00 | APOBEC3F, CASP3, FBXW11, GNAI2, LIMK2,  MAPK1, MAPK11, MAPK9, PPP3CB, RAC2, RAC3, SAMHD1, TRIM5, WEE1 |
| KEGG:05415 | Diabetic cardiomyopathy | 0.07118 | 6.90 | 14.00 | ATP5PO, CTSD, GAPDH, GFPT2, GYS1, MAPK11, MAPK9, NDUFC1, NDUFC2, NOS3, PARP1, PLCB4,  PRKCZ, RAC2 |
| KEGG:05200 | Pathways in cancer | 0.26170 | 5.08 | 27.00 | ADCY9, BIRC3, CASP3, CASP7, DAPK2, EGLN2, GNA13, GNAI2, GSTM1, GSTM4, HSP90AA1, JAK2, MAPK1, MAPK9, MECOM, MET, MGST1, MLH1, MMP1, PDGFRA, PLCB4, RAC2, RAC3, RPS6KA5,  STK4, TXNRD1, VHL |

**Table S2**. Detailed information about antibodies

| Antibody | Vendor | Catalog number | Application |
| --- | --- | --- | --- |
| Anti-AUF1 | Millpore | 07-260 | WB |
| Anti-AUF1 | CST | 12382S | IF, IHC, RIP |
| Anti-AUF1 | Abcam | ab61193 | IF |
| Anti-CagA | Santa Cruz | sc-28368 | WB, IF |
| Anti-p-Tyr | Santa Cruz | sc-7020 | WB |
| Anti-LC3B | Abcam | ab48394 | WB |
| Anti-p62 | Abcam | ab56416 | WB |
| Anti-Flag | Sigma | F1804 | WB |
| Anti-Tubulin | Applygen | C1340-100 | WB |
| Anti-LAMP1 | Santa Cruz | sc-20011 | IF |
| Anti-CTSD | Abcam | ab75852 | WB, IF, IHC |
| Anti-CD81 | Santa Cruz | sc-166029 | WB |

**Table S3**. Sequences of qRT-PCR primers. Gene name Sequence

CTSS (F)5’-TGGATCACCACTGGCATCTCTG-3’ (R)5’-GCTCCAGGTTGTGAAGCATCAC-3’

GALC (F)5’-TATTTCCGAGGATACGAGTGGT-3’ (R)5’-CCAGTCGAAACCTTTTCCCAG-3’

HGSNAT (F)5’-CCGCCACGAGACTTAGACAAA-3’

(R)5’-CAGTAGACGGTCAAGTTGGTC-3’

CTSH (F)5’-TACCTTCGAGGTACTGGTCCCT-3’ (R)5’-GGTGGAGAAAGTCCAGCAACTG-3’

CTSD (F)5’-TGCTCAAGAACTACATGGACGC-3’ (R)5’-CGAAGACGACTGTGAAGCACT-3’

LIPA (F)5’-TCTGGACCCTGCATTCTGAG-3’

(R)5’-CACTAGGGAATCCCCAGTAAGAG-3’

CTSW (F)5’-CGTGACCATCAACATGAAGCCC-3’ (R)5’-CCTCTGACTTGACGCTGCCAAA-3’

AGA (F)5’-CGGAAGTCGAACTTGCCTGT-3’ (R)5’-TCGGTTGCATTCTTAAAGGGC-3’

GAA (F)5’-CTGTTGCTACATCCCTGCAAA-3’ (R)5’-GAGGCGGTTCTCAGTCTCC-3’

GALNS (F)5’-GTGACCTCGGGGTGTATGGA-3’ (R)5’-AAGCCATTGCGGATGGGTAG-3’

CTSE (F)5’-GGACATGATCCAGTTCACCGAG-3’ (R)5’-GAGCCAATGGAGATAGTGCCGA-3’

GBA (F)5’-CATCCGCACCTACACCTATGC-3’ (R)5’-TGAGCTTGGTATCTTCCTCTGG-3’

CTSB (F)5’-GAGCTGGTCAACTATGTCAACA-3’

(R)5’-GCTCATGTCCACGTTGTAGAAGT-3’

HYAL1 (F)5’-CGATATGGCCCAAGGCTTTAG-3’ (R)5’-ACCACATCGAAGACACTGACAT-3’

PPT1 (F)5’-TGTTTTTGGACTCCCTCGATG-3’ (R)5’-CATGCCAGTATTCGGCTTGC-3’

TPP1 (F)5’-CCTCCACACGGTGCAAAAATG-3’ (R)5’-CTCTGCTTGTCGGATGCTCAG-3’

IL-1β (human) (F)5’-ATGATGGCTTATTACAGTGGCAA-3’

(R)5’-GTCGGAGATTCGTAGCTGGA-3’

IL-8 (human) (F)5’-TTTTGCCAAGGAGTGCTAAAGA-3’

(R)5’-AACCCTCTGCACCCAGTTTTC-3’

TNF-α (human) (F)5’-CTCTTCTGCCTGCTGCACTTTG-3’

(R)5’-ATGGGCTACAGGCTTGTCACTC-3’

β-Actin (human) (F)5’-CACCATTGGCAATGAGCGGTTC-3’

(R)5’-AGGTCTTTGCGGATGTCCACGT-3’

IL-1β (mouse) (F)5’-GCAACTGTTCCTGAACTCAACT-3’

(R)5’-ATCTTTTGGGGTCCGTCAACT-3’ IL-8 (mouse) (F)5’-CAAGGCTGGTCCATGCTCC-3’

(R)5’-TGCTATCACTTCCTTTCTGTTGC-3’

TNF-α (mouse) (F)5’-CCCTCACACTCAGATCATCTTCT-3’

(R)5’-GCTACGACGTGGGCTACAG-3’

β-Actin (mouse) (F)5’-GGCTGTATTCCCCTCCATCG-3’

(R)5’-CCAGTTGGTAACAATGCCATGT-3’
